# River Biofilms Microbiome and Resistome Responses to Wastewater Treatment Plant Effluents Containing Antibiotics

**DOI:** 10.3389/fmicb.2022.795206

**Published:** 2022-02-09

**Authors:** Olha Matviichuk, Leslie Mondamert, Claude Geffroy, Margaux Gaschet, Christophe Dagot, Jérôme Labanowski

**Affiliations:** ^1^Institut de Chimie des Milieux et Matériaux de Poitiers, UMR CNRS 7285, University of Poitiers, Poitiers, France; ^2^UMR INSERM 1092, Limoges, France

**Keywords:** antibiotics, antibiotic resistance, resistome, microbiome, wastewater treatment plant (WWTP), river biofilm, network analysis

## Abstract

Continuous exposure to low concentrations of antibiotics (sub-minimal inhibitory concentration: sub-MIC) is thought to lead to the development of antimicrobial resistance (AMR) in the environmental microbiota. However, the relationship between antibiotic exposure and resistance selection in environmental bacterial communities is still poorly understood and unproven. Therefore, we measured the concentration of twenty antibiotics, resistome quality, and analyzed the taxonomic composition of microorganisms in river biofilms collected upstream (UPS) and downstream (DWS) (at the point of discharge) from the wastewater treatment plant (WWTP) of Poitiers (France). The results of statistical analysis showed that the antibiotic content, resistome, and microbiome composition in biofilms collected UPS were statistically different from that collected DWS. According to Procrustes analysis, microbial community composition and antibiotics content may be determinants of antibiotic resistance genes (ARGs) composition in samples collected DWS. However, network analysis showed that the occurrence and concentration of antibiotics measured in biofilms did not correlate with the occurrence and abundance of antibiotic resistance genes and mobile genetic elements. In addition, network analysis suggested patterns of co-occurrence between several ARGs and three classes of bacteria/algae: *Bacteroidetes incertae sedis*, *Cyanobacteria*/*Chloroplast*, and *Nitrospira*, in biofilm collected UPS. The absence of a direct effect of antibiotics on the selection of resistance genes in the collected samples suggests that the emergence of antibiotic resistance is probably not only due to the presence of antibiotics but is a more complex process involving the cumulative effect of the interaction between the bacterial communities (biotic) and the abiotic matrix. Nevertheless, this study confirms that WWTP is an important reservoir of various ARGs, and additional efforts and legislation with clearly defined concentration limits for antibiotics and resistance determinants in WWTP effluents are needed to prevent their spread and persistence in the environment.

## Introduction

Antibiotics (ATBs) are now considered to be ubiquitous contaminants in all kinds of environmental matrices (sediments, soils, water, plants and animals) and present worldwide (Li et al., 2012; Gothwal and Shashidhar, 2015; Aus Der Beek et al., 2016; Patel et al., 2019). Their main origin is related to their excretion and persistence in human or animal feces after administration. In France, there is a whole system of wastewater collection and treatment, either in wastewater treatment plants (WWTP) or, in the case of animal waste (such as manure and slurry) in pits, digesters or methanation units. But all of these treatment methods used are not adapted/optimized to remove antibiotics and other pharmaceuticals (Luo et al., 2014; Kim and Zoh, 2016). Treated effluents, therefore, contain antibiotic residues (and/or their transformation products) which are released into the environment when the effluent is discharged or reused (agricultural spreading). In rivers, these discharges lead to a constant presence of residual concentrations of ATBs and, consequently, exposure of naturally occurring aquatic bacteria that live in structured communities called biofilms (Besemer, 2016).

It has already been found that continuous exposure of natural biofilms to low concentrations of ATBs can lead to various consequences, such as changes in taxonomic composition (Cairns et al., 2018; Danner et al., 2019; Tian et al., 2020), effects on metabolic activity (Crabbé et al., 2019), or even cell death (Friman et al., 2015; Gebreyohannes et al., 2019). However, the most important concern relates to the emergence of antimicrobial resistance (AMR) (Gullberg, 2014; Flemming et al., 2016; Khan et al., 2017). Continuous exposure to low concentrations (sub-minimal inhibitory concentration: sub-MIC) can’t only increase the relative number of antibiotic-resistant bacteria, but also select them for resistance, promote adaptive evolution, and even have unexpected consequences for trophic levels and natural microbial ecosystem functioning (Friman et al., 2015; Hathroubi et al., 2017; Danner et al., 2019).

Although knowledge about the occurrence, transmission, evolution, acquisition, selection, preservation, etc., of AMR and all related theoretical mechanisms have advanced in the last decade, especially through the “One Health” approach, they are still incomplete (Singer et al., 2016; Bengtsson-Palme et al., 2018). The main research has been conducted in the medical/clinical field and has linked the spread of AMR to antimicrobial misuse and overuse as a major factor in the maintenance of AMR. In contrast, the natural variability of resistance genes (RGs), mobile genetic elements (MGEs) and the frequency of their transmission in different types of natural environments are not sufficiently studied (Zhuang et al., 2021). A recent survey of AMR-related research funding showed that only 3% of projects involved research at the environmental scale (Singer et al., 2016). Under natural conditions, the relationship between antibiotic exposure and resistance selection in bacterial communities is less understood. However, awareness of the residual presence of ATBs raises many questions about the role of the environment in the emergence, dissemination and/or maintenance of AMR. Furthermore, it has been shown that resistant bacteria in natural ecosystems have the potential to transfer resistance to pathogenic species through horizontal gene transfer (Stanton et al., 2020). Moreover, there is no simple solution/technology that could be effective in an emergency to clean up all natural environments and stop an explosive spread of AMR (Larsson et al., 2018; Stanton et al., 2020). Thus, repeated/continuous release of antibiotics and resistant bacteria could ultimately pose a significant risk to public health if it makes the environment an uncontrollable reservoir of AMR. The objective of this work was to determine whether current antibiotic contamination of the environment contributes to the spread of AMR in rivers. Therefore, this study examined the relationship between antibiotic concentrations, taxonomic composition and the abundance of antibiotic resistance markers (genes and MGEs) in microbial communities during a 1-year field study.

## Materials and Methods

### Sampling Area and Sample Collection

Natural epilithic biofilm was collected from the Clain river located in the Midwest of France (Supplementary Figure 1). Sampling was conducted within the city of Poitiers at two sites: 1st—upstream (UPS) of the Poitiers WWTP (approximately 2 km from the WWTP discharge area) and 2nd—downstream (DWS) of the WWTP (approximately 5 m from the treated wastewater discharge point). The Poitiers’ WWTP has a nominal capacity of 152,500 inhabitant equivalents (per capita) and treats an average of 20,797 m^3^ of urban wastewater per day (in 2019) (more information provided in the Supplementary Information). Briefly, sterile rocks were placed in the river 5 months before the experiment to allow the formation of natural epilithic biofilms from the river’s microbial communities. At the end of this step, allowing the biofilm to establish, develop, and be exposed, the stones were collected to obtain biofilm samples. A 1-year sampling campaign was conducted from January to December 2018. Beginning from January, several rocks were gradually collected each month, which corresponded to a single bulk sample. Biofilm was carefully scraped off the rocks using a sterile toothbrush that was constantly rinsed with MilliQ grade water. The obtained solution was then carefully centrifuged to remove excess water. The resulting “fresh” biofilms were stored in bottles at −80°C until analyzed.

### Sample’s Preparation and Quantification of Antibiotics

Twenty investigated antibiotics—enrofloxacin (ENR), erythromycin (ERY), levofloxacin (LVX), norfloxacin (NFX), oxytetracycline (OTC), sulfamethazine (SMZ), sulfamethoxazole (SMX), sulfaquinoxaline (SQX), trimethoprim (TMP), ciprofloxacin (CPR), metronidazole (MTZ), roxithromycin (RXM), clarithromycin (CLR), flumequine (FMQ), enoxacin (ENX), tylosin tartrate (T-T), midecamycin (MED), spiramycin (SPR), josamycin (JOS), azithromycin (AZM) (Supplementary Table 1) were purchased from Sigma Aldrich (Buchs, Switzerland). LC/MS-grade methanol, acetonitrile and formic acid were purchased from Carlo Erba Reagents (Val-de-Reuil, France), ultrapure water (>18.2 MΩ cm^–1^) was prepared with the Milli-Q IQ 7000 system (Millipore SAS, Molsheim, France). Individual raw solutions (200 mg/L) were prepared for each antibiotic by dissolving the standard powder in methanol and stored in the dark at −20°C. The standard solution mixtures were prepared at three concentrations (10, 5, 1 mg/L) by diluting the raw solutions with water and then stored in the dark at 4°C. Antibiotics were measured in all the collected biofilms by the procedure described in Aubertheau et al. (2017).

### DNA Extraction and Resistome Analysis

Total bacterial DNA was extracted from freeze-dried biofilms using the Fast DNA^®^ SPIN Kit for Feces (MP Biomedicals, Illkirch, France) according to the manufacturer’s protocol. DNA concentrations were measured using a NanoDrop™ One^C^ Microvolume UV-Vis Spectrophotometer (Thermo Fisher Scientific, United States). Class 1, 2 and 3 integrons were measured by qPCR analysis using the MX3005P real-time detection system (Stratagene^®^) according to the Taqman method described by Stalder et al. (2014). The rest of RGs and MGEs (91 in total) were analyzed using nanoliter-scale quantitative PCR. Between them 66 ARGs, 5 multidrug efflux pumps, 6 heavy metal resistance genes (MRGs), 3 disinfectant RGs and 11 MGEs. The list of RGs and their corresponding primer sequences are given in Supplementary Table 5. The analysis methodology was performed using 96.96 BioMark^®^ Dynamic Array for Real-Time PCR (Fluidigm Corporation, San Francisco, CA, United States). A threshold cycle (Ct) values were extracted using BioMark Real-Time PCR analysis software. The detection limit was set at 20, and the normalized gene abundance was calculated using the 16S rRNA gene abundance according to the following formula: 2^{−[*Ct*(*RG*) − *Ct*(16*S* rRNA)]}^. The analysis procedure is described in more detail by Buelow et al. (2018, 2020).

### Microbial Community Analysis

Part of each sample was sent to a European sequencing platform (MACROGEN, EUROPE B.V., Amsterdam, Netherlands^1^) to perform microbial community analysis. There, total DNA was extracted using the PowerSoil^®^ DNA Isolation Kit (QBioGene, Solon, OH, United States) following the manufacturer’s instructions. Sequencing samples were prepared according to Illumina 16S Metagenomic sequencing library protocols. The hypervariable V3-V4 16S region of the bacterial gene was targeted by the universal bacterial primers PCR 341F and 805R (Herlemann et al., 2011). Gene amplification and sequencing [using the Illumina platform (Illumina Inc., San Diego, CA, United States)] were performed by Macrogen Ltd. (Seoul, Korea), with the Herculase II Fusion DNA Polymerase Nextera XT Index kit V2 (library kit) and following the 16S metagenomics sequencing library preparation protocol^2^.

Assembly was performed using the FLASH (Fast Length Adjustment of SHort Reads) software tool to merge paired-end reads. The raw data were processed using CD-HIT-OTU. Short reads were filtered out and ultra-long tails were trimmed. The filtered reads were clustered at 100% identity (using CD-HIT-DUP) and chimeric reads were identified. Secondary clusters were then joined to primary clusters and noise sequences from these clusters were removed. Finally, the remaining representative reads from non-chimeric clusters were clustered using a greedy algorithm into an operational taxonomic unit (OTU) (at the species level). OTU clustering was used in the *de novo* OTU selection method (CD-HIT). Taxonomic distribution and diversity statistics were performed using Qiime 2 version 2019.10 Taxonomic software using the NCBI database (BLAST, NCBI_16S_20210518). Classified OTUs were determined at ≥ 98% sequence homology and converted to percentages (relative abundance) to determine the representation of each microbe among treatments. OTUs with a relative abundance of less than 0.001% were excluded. Diversity alpha (Shannon, Simpson, and Chao1), sparsity analysis, and beta diversity index (Bray–Curtis similarity) were evaluated using Qiime2 software (version 2019.10).

### Statistical Analysis

Discriminant function analysis (DFA) was used to examine whether antibiotic concentrations and the abundance of resistance genes differ significantly between the two sites studied (UPS and DWS). This analysis was performed using XLSTAT. Non-metric multidimensional scaling (NMDS), Goodness-of-fit test, Mantel test, Adonis test and Procrustes analysis were conducted based on Bray–Curtis dissimilarity matrices on RStudio (version 4.0.5) using package “Vegan” for ecological statistical analysis. Based on the NMDS results, Procrustes analysis was performed using the protest function with 999 permutations to verify the significance. To assess the effect of different ATB concentrations on RGs abundance, the quantitative data were converted into binary data. To do this, we compared the obtained concentrations with the minimal selective concentration (MSC) and the minimal inhibitory concentration (MIC), and according to whether this concentration corresponded to one or another of the mutant selection window (MSW) hypothesis ranges, this value was converted to 1, or 0. Thus, three data sets were prepared that separated samples with concentrations less than MSC, greater than MIC and those that fell within MSW. These data were then used to construct a network analysis. The network analysis between ATBs and RGs was based on the dissimilarity coefficient calculated from the binary data using XLSAT. Similarly, Pearson’s dissimilarity coefficient from the quantitative data was calculated between the most abundant classes of bacteria and RGs. The network visualization, as well as the calculation of the degree of nodes and edges betweenness, were performed in Cytoscape 3.8.2 software, where only the dissimilarity coefficients > 0.7 were displayed.

## Results and Discussion

### Temporal Dynamics of Antibiotics, Resistome and Microbiome in the Clain River Biofilms

**FIGURE 1:**
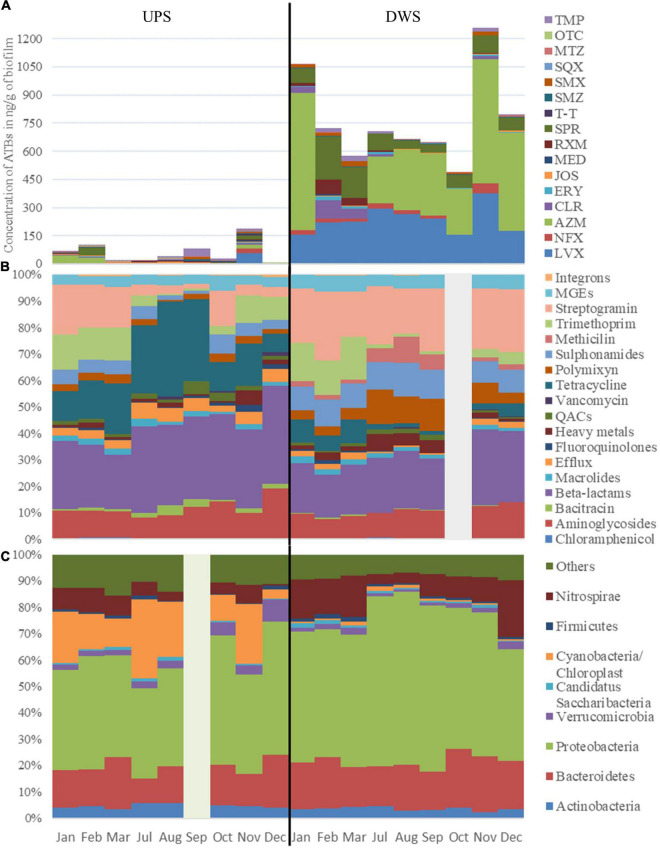
Dynamic of panel **(A)** ATBs concentration in ng/g of biofilm, of panel **(B)** cumulative abundance of the resistome, grouped into gene classes and of panel **(C)** most abundant bacterial phyla in biofilms collected UPS and DWS of the WWTP. Due to analytical problems results for October (resistome composition) and for September (taxonomic composition) are not included.

The results of the 1-year survey showed the presence of ATBs from different families in the biofilms. Between 19 and 20 antibiotics (out of 20 analyzed) were detected in Clain river biofilms collected upstream (UPS) and downstream (DWS) of the WWTP, respectively (Figure 1A and Supplementary Figure 2). Only CPR was not detected at the UPS samples. The values obtained show that the total median ATB concentrations DWS exceed the corresponding concentrations measured UPS (220 vs. 851 ng/g of biofilm). Statistical comparison of the values by DFA showed that this difference is significant (Wilks’ lambda = 0.000002770 and *p*-value = 0.005—Table 1) between the biofilms collected from both sites. The difference is mainly due to twelve of the 20 ATBs studied, whose concentrations were found to be significantly different (Supplementary Table 2). The results also revealed differences in contamination depending on the sampling periods at the same site. In DWS biofilms, CLR and SPR concentrations increased in February and March, while AZM was not detected in the same period, although it was the main ATB detected in other sampling months. At UPS, the distribution of ATBs is much more variable each month, with no trend or pattern. In November the concentration of almost all ATBs was significantly higher than during other months. Some previous works had already pointed out the presence of similar concentrations of pharmaceuticals (including AZM, CPR, CLR, LVX, NFX, SMX and TMP) in river biofilms (Aubertheau et al., 2017; Auguet et al., 2017; Carles et al., 2021; Valdés et al., 2021) and also observed variations in the distribution of these compounds over time (Valdés et al., 2021).

**TABLE 1:** Results of the statistical comparison of the concentration of ATBs and normalized abundance of RGs measured in biofilms collected UPS and DWS from the WWPT.

	Comparison of ATBs concentration	Comparison of normalized abundance of resistance genes and MGEs
Wilks’ lambda	0.000002770	0.00000648
F (Observed value)	22,566.915	10,283.206
F (Critical value)	246.464	245.950
*p*-value	0.005	0.008
Alpha	0.05	0.05

It is well known that ecosystems affected by human activities are generally the most polluted by ARGs (Chu et al., 2018; Zhao et al., 2018; González-Plaza et al., 2019; Buelow et al., 2020; Kunhikannan et al., 2021). Zhuang et al. (2021) conducted a large analysis of scientific publications on the distribution and diversity of ARGs published over the past 30 years (from 1990 to 2020). The results of the nearly 10,000 publications they analyzed showed that the most contaminated habitats for ARGs (after hospitals and farms) were sewage treatment plants and surface water, with little difference between them. The resistome study of UPS samples showed that even without direct exposure to WWTP discharge, river biofilms contain significant amounts of RGs. Anthropogenic expansion means that even areas upstream of cities are likely to be impacted (to a lesser extent) by different discharges (e.g., agricultural, on-site sanitation) that maintain/contribute to the presence of RGs in river biofilms. If the ARGs genetic diversity is close between UPS and DWS, the location directly downstream of discharge clearly maintains a greater potential of selection pressure. Thus, river biofilms are reservoirs with the potential to acquire and maintain resistance to antibiotics, which, however, is characterized by their exposure degree.

The study of the resistome-mobilome showed that the differences in the abundance of resistance genes and MGEs measured in biofilms collected from both sites are statistically significant (Wilks’ lambda **=** 0.00000648, *p*-value = 0.008) (Table 1). The unidimensional test of equality showed (Supplementary Table 3) that the normalized abundance of 31 of the 57 RGs and MGEs, which were quantified in more than three samples at both sites, differed significantly between the biofilms collected UPS and DWS. The normalized abundances, shown in Figure 1B and Supplementary Table 7, demonstrate the difference in gene abundance distribution (in %) between the two sites. More tetracycline, beta-lactam and efflux RGs were detected in UPS but fewer MGEs, streptogramin, sulfonamides and polymyxin compared to DWS. The results also show that the gene distributions evolve slightly by periods of a few months over the 1-year monitoring period. Thus, at the UPS site, a significant proportion of streptogramin and trimethoprim RGs is observed from January to March, then their abundance decreases strongly in favor of tetracycline RGs from July to September, but from October to December their proportion increases again. At the DWS site, resistome analysis shows a significant proportion of trimethoprim and tetracycline RGs from January to March, then, from July to September, their abundance decreases in favor of methicillin and polymyxin RGs, after which methicillin abundance decreases, and beta-lactams increase in November and December.

To complete the monitoring of biofilms, the taxonomic composition of biofilms was analyzed in parallel with the above-mentioned analyses. The results show very little variation in taxonomic composition (by Phylum level) between samples collected at the same site for 1 year. At the UPS site, the changes were associated mainly with the number of *Cyanobacteria/Chloroplast* and *Nitrospirae* (the number of both decreased in December) and *Verrucomicrobia* (increased in December). At the DWS site in July and August there was a decrease in the number of *Nitrospirae*, but an increase in *Proteobacteria*. The taxonomy diversity also reveals varieties of different species and much larger numbers of *Cyanobacteria*/*Chloroplast* and *Verrucomicrobia* in the UPS-collected samples (Figure 1C). Biofilms collected DWS contain visibly more *Nitrospirae* (*Nitrospira*) and *Proteobacteria* (*Alpha*, *Beta-, Delta*- and *Gamma*- *proteobacteria*), as well as *Bacteroidetes* (*Bacteroidetes_incertae_sedis, Flavobacteria and Sphingobacteriia*) (Supplementary Figure 3). The phylum *Proteobacteria* consists of Gram-negative bacteria that include a wide variety of pathogens. The abundance of this phylum in WWTPs discharges, in sediments and activated sludge is common (Liang et al., 2009; Atashgahi et al., 2015; Zhang et al., 2019), since *Proteobacteria* adapted to low-nutrient conditions and are very common in the mammalian gastrointestinal tract (Moon et al., 2018). The presence of *Nitrospirae* can be explained by the fact that effluents of WWTPs are rich in nitrate, as nitrification is a common process in the biological removal of nitrogen in WWTPs (McCarty, 2018). These results correlate with previous studies that analyzed microbial communities of river bacterioplankton in Australia, plankton with sediments in an urbanized river of Brazil and biofilm collected in drinking water distribution system in China where *Cyano-, Proteo* -, and *Actinobacteria* were dominant phyla (Carney et al., 2015; Köchling et al., 2017; Chen et al., 2020). A metagenomic study of artificially grown biofilms in a Chinese urban river showed the same results (Cai et al., 2018).

The taxonomic analysis also pointed out 17 different genera of pathogenic or opportunistic pathogens at both sites, as shown in Supplementary Figure 4. *Aeromonas*, *Clostridium* and *Pseudomonas spp*. were found to be the most abundant potentially pathogenic genera in studied samples. The number of pathogenic bacteria can be particularly significant DWS from WWTPs, which are important sources of pathogen releases since traditional treatment usually has low efficiency for pathogen removal (Aghalari et al., 2020; Mao et al., 2021). In addition, biofilm formation is characteristic of most bacterial pathogens (for about 80%) (Balcázar et al., 2015; Khatoon et al., 2018; Chen et al., 2020).

### Incidence of Season and Exposure

**FIGURE 2:**
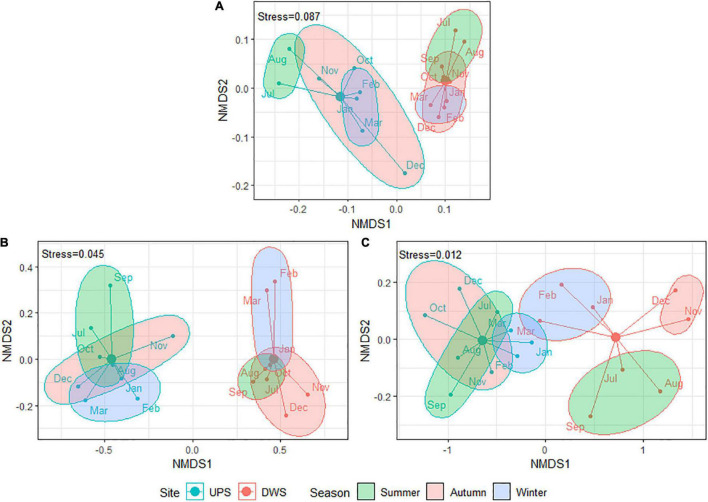
Bray–Curtis based NMDS plots showing difference between square root transformed data of panel **(A)** taxonomic composition (Goodness-of-fit: R^2^ = 0.61, *p*-value < 0.001; Adonis test: R^2^ = 0.53, *p*-value = 0.002; Stress = 0.087), panel **(B)** ATBs composition (Goodness-of-fit: R^2^ = 0.83, *p*-value < 0.001; Adonis test: R^2^ = 0.68, *p*-value < 0.001; Stress = 0.045) and panel **(C)** resistome composition (Goodness-of-fit: R^2^ = 0.68, *p*-value < 0.001; Adonis test: R^2^ = 0.51, *p*-value < 0.001; Stress = 0.013) between studied biofilms by sampling site and season [samples were divided into three corresponding seasons: summer (June—August), autumn (September—November), and winter (December—February)]. Due to analytical problems results for September (taxonomic composition) and October (resistome composition) are not included. The samples were named by the month of sampling, but they actually reflect the 30 days prior to the sampling date.

A NMDS based on Bray–Curtis dissimilarity matrices was carried out to examine the dissimilarities in ATBs concentration, resistome abundance and microbial community composition among the biofilms collected over 1 year (samples were grouped into three seasons). The resulting ordination plots demonstrate that the differences in microbial taxonomic composition (Figure 2A) and ATBs content (Figure 2B) between the biofilms for both studied sites are statistically significant (confirmed by Goodness-of-fit and Adonis tests). This finding reveals that exposure to WWTP effluent alters taxonomic diversity and increases antibiotic contamination of the biofilm. However, likely, some of the species identified are not living bacteria, but rather remnants of dead bacteria dumped by the WWTP.

Resistome composition also significantly differed by location (Figure 2C). In addition, Goodness-of-fit and Adonis tests revealed statistically significant changes in resistome and taxonomic composition in the biofilms of both sites (UPS and DWS) as a function of the season (*p*-value = 0.025/0.005 for resistome and *p*-value = 0.014/0.019 for microbiome collected UPS and DWS, respectively). At the same time, seasonal changes in ATB concentration were observed only in samples collected DWS (*p*-value = 0.003). Note that the NMDS dissimilarity plots shown in Figure 2 do not give a good indication of the effect of seasons on the distribution of samples, since these are two-dimensional plots.

This finding is consistent with other studies showing changes in river bacterial communities with changes in water temperature (Kaevska et al., 2016; Wang et al., 2021). Several studies have also reported seasonal variations in resistome composition in untreated hospital wastewater in France and Turkey (Buelow et al., 2020; Gönder et al., 2021), in sewage wastewater in Germany (Caucci et al., 2016), in WWTP effluents and river water in Poland (Harnisz et al., 2020). This effect may be related to the concentration of ATBs in the wastewater, which was also reported to be seasonally dependent (Zhang et al., 2015; Gönder et al., 2021). For example, the seasonality of ARGs in wastewater observed by Caucci et al. (2016) was related to the level of antibiotic prescribing in the sampling region. Seasonality in ATB use has already been reported in Europe and the United States, where the highest ATB consumption occurred during the cold period (winter and spring) (Elseviers et al., 2007; Suda et al., 2014).

### What Potential Risk Does River Biofilm Contamination Pose?

**FIGURE 3:**
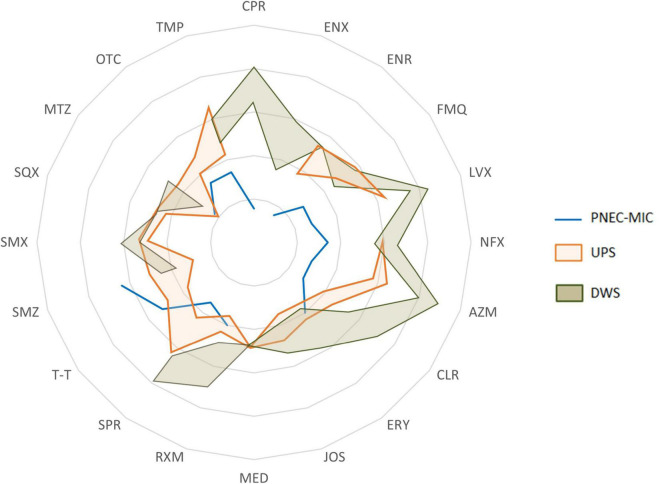
A spider chart showing the range (from min to max) of antibiotics concentrations measured in biofilms collected upstream (UPS) and downstream (DWS) from the WWTP compared to PNEC-MIC.

The permanent coexistence of antibiotics (and probably other co-selectors), microorganisms (including pathogens) and antibiotic resistance determinants (ARGs, MGEs, etc.) makes biofilms potentially favorable micro-environments for the selection, co-selection, transmission and spread of RGs or resistant bacteria. To assess the extent of ATB concentrations in biofilms, the concentrations found were compared with the corresponding predicted no-effect concentrations for the resistance selection (PNEC-MIC) (Figure 3). The comparison showed that the concentrations measured in biofilms could theoretically cause the maintenance and enrichment of existing resistant strains and even lead to the selection of new resistant mutants.

Many studies confirm that anthropogenic pressure contributes to the increase of antibiotic resistance markers (genes and bacteria) in the environment. However, selection and maintenance are not necessarily due to the effect of ATB presence alone. The enrichment of ARGs can be due to cross-resistance effects or to the presence of co-selectors, which are widely present in anthropized ecosystems. Indeed, several studies have proven that MRGs and MGEs can contribute to the maintenance and proliferation of ARGs (Wales and Davies, 2015; Pal et al., 2017; Partridge et al., 2018; Yang et al., 2018; Glibota et al., 2020). Based on this, several options for the enrichment of ARBs in human-exposed environments are possible: (1) selection under high antibiotic pressure or (2) enrichment by MGEs carrying RGs to various antimicrobial agents (including antibiotics) and selective pressure from a cocktail of contaminants (i.e., co-selection).

Some previous works had already pointed out the presence of pharmaceuticals (including AZM, CPR, CLR, LVX, NFX, SMX and TMP) (Aubertheau et al., 2017; Auguet et al., 2017; Carles et al., 2021; Valdés et al., 2021) and MGEs (integrons) in similar samples (Aubertheau et al., 2017; Auguet et al., 2017; Chen et al., 2020; Li and Zhang, 2020). However, it was not clear how the presence of integrons can be linked to the presence of ATBs, as integrons also contain genes other than ARGs or encoding proteins of unknown function.

### Relationship Between Resistome and Antibiotics

**FIGURE 4:**
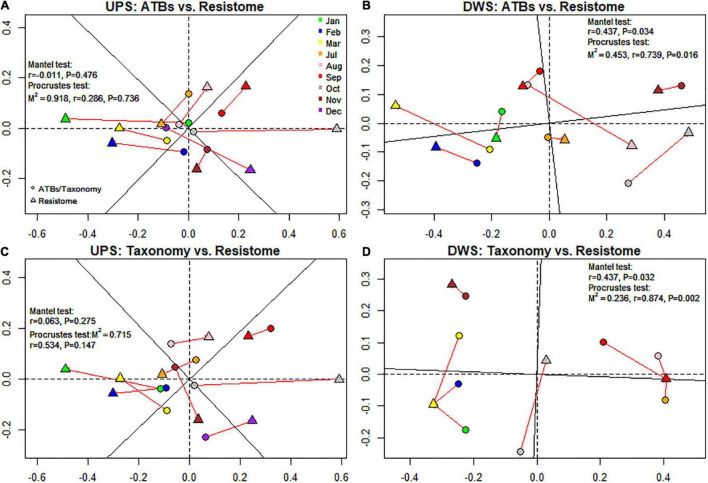
NMDS-based procrustean analysis showing correlation between resistome and ATB content **(A,B)** and resistome and microbial community **(C,D)** for both studied sites. M^2^, sum of squares, r, correlation in a symmetric Procrustes rotation, P, *p*-value (significance).

Procrustes analysis was performed to find out whether the occurrence of RGs is related to the presence of ATBs. This assay was used to see if the total number of RGs was related to the amount of antibiotics measured in biofilms. For this purpose, the results of NMDS ordination based on the dissimilarity matrix were used. The Mantel test was also performed to confirm the results of the previous one. As shown in Figure 4A, the results of the Mantel and Procrustes analyses showed no correlation between the matrices. In contrast, for samples collected DWS (Figure 4B), the resistome dissimilarity matrix has a significant positive relationship with ATBs (M^2^ = 0.45, *r* = 0.74, *p*-value = 0.016 for Procrustes analysis; *r* = 0.44, *p*-value = 0.034 for Mantel analysis).

However, while Procrustes analysis can provide a global view of the overall correlations between ATBs and resistome, it cannot clarify the relationships between individual variables. To examine the relationships between each ATB and its corresponding RG, we assessed the correlation using a network analysis, which can be an effective complement to general correlation information obtained by Procrustes analysis. The patterns of co-occurrence were investigated using network analysis based on strong (*r* > 0.7) correlations. To better study the effect of different ATBs concentration on AMR development, the idea of the MSW hypothesis was applied (Drlica and Zhao, 2007; Gullberg et al., 2011). For this purpose, ATB concentrations were sorted into three groups: those less than the MSC, those between the MSC and the MIC (which means that they fall within the MSW range), and those greater than the MIC. Accordingly, a biofilm network analysis was performed for each of the three cases (Supplementary Figures 5–7).

To simplify the reading of complex network graphs with multiple connections that overlap each other, the results, we are most interested in, have been summarized in Table 2. A synthesis of the results presented in this table shows that the correlation between ATBs and RGs can be divided into several groups. For the UPS-collected biofilms four groups can be distinguished. The first group (LVX, AZM and ENR) corresponds to the ATBs that have been found to correlate with at least one of the associated RG in each measured concentration range. The second group (NFX, FMQ and CLR) corresponds to the ATBs that are present in two concentration ranges (at < MSC and in MSW) and only when present in MSW they were found to be associated with the relevant RGs. The third group (T-T, RXM, ERY and SMX) corresponds to the ATBs that are quantified in all samples only at concentrations < MSC and were associated with several RGs. The fourth group consists of ATBs such as TMP, OTC and MTZ that have not been correlated with any of the associated RGs, although they have been detected in concentrations consistent with those that may be responsible for resistance selection. Finally, several of the listed ATBs (except SPR, NFX, TMP, OTC and MTZ) were found correlated with a group of the same RGs, without matching logic.

**TABLE 2:** Summary table of correlation between resistome and ATBs in concentrations corresponding to the three ranges of the MSW hypothesis.

Antibiotic concentration/Related RGs	UPS			DWS		
	< MSC	MSC-MIC	> MIC	< MSC	MSC-MIC	> MIC
CPR	–	–	–	–	+	+
	–	–	–	–	*qnr*A	*qnr*A
ENR	+	–	+	+	–	+
	*qnr*A, *qnr*S	–	*qnr*B, *qnr*C, *mfs*A	*qnr*A, *qnr*S, *mfs*A	–	*qnr*A, *qnr*S, *mfs*A
FMQ	+	+	–	–	+	–
	–	*qnr*B, *qnr*C, *mfs*A	–	–	*qnr*S	–
LVX	–	+	+	–	+	+
	–	*qnr*A, *qnr*S	*qnr*A, *qnr*S	–	*qnr*A	*qnr*A
NFX	+	+	+	–	+	+
	–	*mfs*A		–	*qnr*A	*qnrA, mfsA*
AZM	–	+	+	–	+	+
	–	*ermC, ermX, macB, mefA_10*	*ermC, mefA_10*	–	*erm*C	*erm*C
CLR	+	+	–	–	+	+
	–	*macB, mefA_10, ermC*	–	–	–	–
ERY	+	–	–	–	+	+
	*erm*X, *mac*B	–	–	–	–	–
RXM	+	–	–	+	–	+
	*erm*C, *erm*X, *mac*B, *mef*A_10	–	–	*mef*A_10	–	–
SPR	+	+	–	–	+	+
	–	–	–	–	*erm*C, *erm*X, *erm*Y, *mac*B	–
T-T	+	–	–	+	–	–
	*erm*C, *erm*X, *mac*B, *mef*A_10	–	–	–	–	–
SMX	+	–	–	+	–	–
	*sul*1, *sul*A	–	–	*sul*1, *sul*A	–	–
MTZ	+	+	–	–	+	–
	–	–	–	–	–	–
OTC	+	+	–	+	–	–
	–	–	–	*tet*B, *tet*Q	–	–
TMP	+	+	+	+	+	+
	–	–	–	–	–	–

*The first column lists all antibiotics with already defined concentration ranges for the mutant selection window (MSC and MIC). There are two rows opposite each antibiotic. The top row shows the concentration range in which the antibiotic was measured in the biofilm (highlighted in green), and the bottom row shows whether the network analysis showed a link between that antibiotic and the corresponding RGs, and if so, with which gene.*

For the biofilms collected DWS, the distribution of ATBs by groups is slightly different. CPR, NFX, LVX and AZM were found in concentrations falling in two out of three ranges (MSW and > MIC) and in both cases, they were associated with at least one corresponding RG. RXM, SMX, and OTC were found to be associated with the corresponding RGs at concentrations < MSC, while FMQ and SPR at concentrations < MIC. ENR was detected in concentrations either below or above the MSW, but in all cases was associated with several fluoroquinolones’ RGs. The remaining ATBs (CLR, ERY, TMP, MTZ and T-T) were not correlated with any of the corresponding RGs in any case.

The results of the network analysis (DWS) show correlations, but these are more related to co-occurrence than to pressure/selection mechanisms (based on the MSC and MIC). Thus, RXM and SPR correlate with corresponding RGs at concentrations < MSC or in the MSC-MIC range (in the case of SPR), but no longer correlate at concentrations > MIC, where resistant strains generally dominate. Most likely, the explanation can be found in the hypothesis that the genes measured in the DWS-collected biofilms originate from genetic residues captured/trapped by the biofilm matrix. Indeed, DWS biofilms contain MGEs and cell-free DNA that play an important role in horizontal gene transfer (HGT) and the spread of ARGs in bacterial communities (Woegerbauer et al., 2020). In addition, because the MSC and MIC values were derived from assays performed on pure cultures, these values were compared with the predicted no-effect concentrations for resistance selection (PNEC^R^) published by Murray et al. (2020). In this paper, the authors presented data on experimentally derived selective effect concentrations of several antibiotics (five of the 20 analyzed in our study) from studies of reduced growth of a complex bacterial community in wastewater and changes in the abundance of class 1 integrons. For two antibiotics (TMP and CPR), the MSC and MIC values we used were lower than the PNEC^R^ values published by Murray et al. (2020) (Supplementary Table 4). For the other antibiotics (ERY, CLR, and AZM), the PNEC^R^ values are much higher than the MIC. However, when looking at Table 2, for two antibiotics (ERY, CLR) no correlations were found with the corresponding genes at concentrations greater than MIC, while for AZM correlations were observed at concentrations > MSC and > MIC. Thus, based on the literature data (obtained from both pure cultures and multispecies communities), it can be concluded that there is no correlation between the concentrations of various antibiotics theoretically favorable for the selection of ARGs/MGEs in river biofilms, suggesting that the occurrence of ARGs and MGEs is not related to the antibiotic concentrations measured in river biofilms (no matter exposed or not to the WWTP discharges).

The emergence of resistance genes may be related to their persistence, since resistance acquired by co-selection can persist even at low concentrations of ATB (which has already been observed in various freshwater ecosystems). There is even considerable evidence in the literature for the persistence of ARGs in environments without ATB or located far from sources of antibiotic release and therefore containing very low concentrations (Andersson and Hughes, 2011; Proia et al., 2016; Lopatkin et al., 2017).

### Relationship Between Resistome and Microbiome

The relationship between resistome and microbiome was also investigated. The results of the Procrustean analysis revealed a significant correlation pattern of resistome with the microbial communities of the biofilms collected DWS of the WWTP (M^2^ = 0.24, *r* = 0.87, *p*-value = 0.003 for Procrustes analysis; *r* = 0.44, *p*-value = 0.032 for Mantel analysis), whereas no correlation was observed in samples collected UPS (Figures 4C,D).

The relationship analysis between resistome and microbiome was supplemented by network analysis, as previously done for resistome and ATB concentrations. Nineteen classes of bacterial species, most common in the samples analyzed, were selected for this analysis (Supplementary Figure 8 and Table 3). The results show that 10 classes of bacteria could be potential carriers of 35 and 7 RGs/MGEs in biofilms collected UPS and DWS, respectively. Among these 10 classes, *Nitrospira* and *Bacteroidetes incertae sedis* probably carry the greatest number of diverse genes (27 and 14, respectively) in the UPS-collected biofilms. *Nitrospira* was correlated with almost all groups of RGs analyzed: chloramphenicol RGs (*cat* and *cat*B3), aminoglycoside RGs [*aad*B, *aph(3*′*)-III, AAC(3)-Ib*], bacitracin RG (*bacA_1*), beta-lactam RGs (*bla*Per-1, *bla*_IMP_, *bla*_TEM_, *bla*_KPC_, *bla*_DHA_ and *cbl*A), macrolide RGs (*erm*C, *erm*Y, *erm*X), one multidrug RG (*mdt*L), MRGs (*cus*F, *cop*D), RG of quaternary ammonium compounds (QACs) (*qac*A), methicillin RG (*mec*A), trimethoprim RG (*dfr*F), polymyxin RG (*arn*A), sulfonamide RGs (*sul*1, *sul*A), transposase genes (*IS6100, ISS1N*) and streptogramin RG [*vat*(A)]. *Bacteroidetes incertae sedis* were associated with beta-lactam RGs (bla_KPC_, bla_DHA_ and *cbl*A), chloramphenicol RGs (*cat* and *cat*B3), methicillin RG (*mec*A), trimethoprim RG (*dfr*F), polymyxin RG (*arn*A), transposase genes (*IS6100, ISS1N*), class 1 integron integrase (*Intl*1), ERY RG (*ermC*), sulfonamide RG (*sul*1) and streptogramin RG [*vat*(A)]. *Chloroplast* was a possible host of two beta-lactam RGs (*bla*_CTX–M_, *bla*_IMP_), multidrug RGs (*mdt*L, *tol*C), one macrolide RG (*mef*A_10), cooper RG (*cus*F) and QACs RG (*qac*A). Finally, *Verrucomicrobiae* was found to be correlated with class 3 integron integrase (*Intl*3), vancomycin RG (*van*A) and aminoglycoside RG [*aac(6*′*)-IIa*], and *Acidobacteria_Gp6* with *TTV*. Other works have already published evidence on the close association between resistome and microbiome in different compartments (wastewater, soil, and human intestinal tract) (Zhao et al., 2018). However, one cannot be certain that the measured abundance of resistance genes is solely due to the taxonomic composition of biofilms. The correlation between certain bacterial species and RGs could mean that the presence of certain genes is due to and/or favored by the presence of particular strains; or that there is co-selection by MGEs carrying genes encoding resistance to more than one type of antimicrobial agent (e.g., disinfectants and antibiotics); or that it is simply a co-occurrence.

**TABLE 3:** Summary table of correlation between resistome and microbial communities.

Microbial communities		Genes Biofilms	
Phylum	Class	UPS	DWS
Acidobacteria	*Acidobacteria_Gp6*	*TTV*	*aac(6*′*)-IIa*
Actinobacteria	*Actinobacteria*	–	–
Bacteroidetes	*Bacteroidetes_ incertae_sedis*	*cat, catB3, cbl*A, *bla_DHA_, bla_KPC_, erm*C, *arn*A, *sul*1, *mec*A, *dfr*F, *vat*(A), *ISS1N, IS6100, Intl*1	–
	*Flavobacteriia*	–	–
	*Sphingobacteriia*	–	–
Gemmatimonadetes	*Gemmatimonadetes*	*erm*C	–
Proteobacteria	*Alphaproteobacteria*	–	–
	*Betaproteobacteria*	–	–
	*Deltaproteobacteria*	–	–
	*Gammaproteobacteria*	–	*bla_VIM_, acr*A, *mdf*F, *cus*F, *dfrA27*
Verrucomicrobia	*Opitutae*	–	*ISS1N*
	*Subdivision3*	–	–
	*Verrucomicrobiae*	*aac(6*′*)-IIa, van*(A), *Intl*3	–
Candidatus Saccharibacteria	*Candidatus Saccharibacteria*	–	*aac(6*′*)-IIa*
Chloroplast	*Chloroplast*	*bla_CTX–M_, bla_IMP_, mefA_10, tol*C, *mdt*L, *cus*F, *qac*A	–
Cyanobacteria	*Cyanobacteria*	–	*arc*A, *cus*F
Firmicutes	*Bacilli*	–	–
	*Clostridia*	–	–
Nitrospirae	*Nitrospira*	*cat, cat*B3, *aph(3′)-III, aad*B, *AAC(3)-Ib, bacA_1, cbl*A, *bla*_TEM_, *bla*_IMP_, *bla*_DHA_, *bla*_KPC_, *blaPer-1, ermC, ermY, ermX, mdtL, cusF, cop*D, *qac*A, *arn*A, *sul*1, *sul*A, *mec*A, *dfr*F, *vat*(A), *ISS1N, IS6100*	–

As for the samples collected DWS, only *Gammaproteobacteria* showed the correlation pattern with several resistance genes such as beta-lactam (*bla*_VIM_), cooper RG (*cus*F), multidrug RGs (*arc*A and *mdf*F) and TMP RG (*dfr*A27). Other bacterial phyla had links to only one or two RGs. The lack of significant (non-random) links between bacteria and antibiotic resistome DWS from the WWTP once again indicates that the measured abundance of RGs is likely to be unrelated to the presence of ATBs.

Bottery et al. (2020) examined in detail AMR in bacterial communities and argued that the evolution of bacterial resistance is not only the result of antibiotic exposure but also their combined effects along with bacterial interactions within the communities. But in this study, a comparison of the resistome and microbiome of river biofilms proved difficult to identify specific bacterial communities associated (hosts) with ARGs. Bottery et al. (2020) claimed that bacteria living in biofilms have more favorable conditions compared to free-living species (planktonic lifestyle) because the sessile lifestyle increases the probability of compensatory mutations, which allows stepwise generation of low-cost resistance mutations. The role of compensatory mutations in the acquisition of a low-cost resistance mutation has also been supported by some other researchers (Andersson and Hughes, 2011; Dunai et al., 2019). Moreover, Gullberg et al. (2011) argued that the selection of low-cost and no-cost resistance mutations is favored by sub-MIC antibiotic concentrations.

On the other hand, it is important to remember that in this study ARGs, MGEs, and MRGs abundance had been evaluated during point measurements Various parameters and processes, such as physical, chemical (sorption/desorption, degradation), or biological (reproduction, aging, biodegradation), can influence and change the number of genes. Some ATBs are known to be able to persist for a very long time in river sediments. Several works show that antibiotics (Hektoen et al., 1995) and ARGs (Muziasari et al., 2014; Devarajan et al., 2015) can persist in deep layers of sediments. Likewise, many factors suggest the long-term persistence of ARGs *in vivo*, since, in the absence of fitness costs, resistant mutants will persist even when antibiotic use is discontinued. Andersson and Hughes (2011) cited several examples of studies proving that antibiotic resistance persists for many years before returning to its original state prior to ATB exposure. However, results obtained by Dunai et al. (2019), who tested the effectiveness of compensatory evolution in multidrug-resistant *E. coli* under antibiotic-free conditions, showed that such conditions can lead to reduced resistance to certain but not all ATBs. Nevertheless, a similar test on biofilm communities in addition to single-cell cultures would have been necessary to confirm these observations, since this lifestyle is more common in most natural ecosystems.

## Conclusion

It appears that the presence of resistance markers in environmental bacteria is a complex process that cannot be explained by considering ATB concentration as the main determinant. The results obtained in this study raises many rather fundamental questions about why the antibiotic dose/resistance selection mechanism in biofilms is ineffective. The complex matrix of environmental biofilms likely affects the bioavailability and bioaccessibility of ATBs, which may limit their impact on microbial communities. Specific studies are needed to better characterize the interaction of ATB with biofilm components, especially at the scale of the microenvironment within the biofilm.

## Data Availability Statement

The original contributions presented in the study are included in the article/Supplementary Material, further inquiries can be directed to the corresponding author/s.

## Author Contributions

OM participated in sampling campaigns, performed analytical analyses and sample preparation for metagenomic studies and resistome analysis, performed statistical analyses, and drafted the manuscript. MG participated in the metagenomic studies. JL and LM conceived, funded and coordinated the study, organized and participated in the sampling campaigns, and reviewed and edited the manuscript. CD and CG conceived and coordinated the study, reviewed and edited the manuscript. All authors contributed to the article and approved the submitted version.

## Conflict of Interest

The authors declare that the research was conducted in the absence of any commercial or financial relationships that could be construed as a potential conflict of interest.

## Publisher’s Note

All claims expressed in this article are solely those of the authors and do not necessarily represent those of their affiliated organizations, or those of the publisher, the editors and the reviewers. Any product that may be evaluated in this article, or claim that may be made by its manufacturer, is not guaranteed or endorsed by the publisher.

## Footnotes

1
https://www.macrogen.com/

2
https://support.illumina.com/documents/documentation/chemistry_documentation/16s/16s-metagenomic-library-prep-guide-15044223-b.pdf

## References

[B1] Aghalari Z. Dahms H. U. Sillanpää M. Sosa-Hernandez J. E. Parra-Saldívar R. (2020). Effectiveness of wastewater treatment systems in removing microbial agents: a systematic review. *Global. Health.* 16:13. 10.1186/s12992-020-0546-y 32013988 PMC6998187

[B2] Andersson D. I. Hughes D. (2011). Persistence of antibiotic resistance in bacterial populations. *FEMS Microbiol. Rev.* 35 901–911. 10.1111/J.1574-6976.2011.00289.X 21707669

[B3] Atashgahi S. Aydın R. Dimitrov M. R. Sipkema D. Hamonts K. Lahti L. (2015). Impact of a wastewater treatment plant on microbial community composition and function in a hyporheic zone of a eutrophic river. *Sci. Rep.* 5:17284. 10.1038/srep17284 26607034 PMC4660315

[B4] Aubertheau E. Stalder T. Mondamert L. Ploy M. C. Dagot C. Labanowski J. (2017). Impact of wastewater treatment plant discharge on the contamination of river biofilms by pharmaceuticals and antibiotic resistance. *Sci. Total Environ.* 579 1387–1398. 10.1016/j.scitotenv.2016.11.136 27913024

[B5] Auguet O. Pijuan M. Borrego C. M. Rodriguez-Mozaz S. Triadó-Margarit X. Giustina S. V. D. (2017). Sewers as potential reservoirs of antibiotic resistance. *Sci. Total Environ.* 605–606 1047–1054. 10.1016/J.SCITOTENV.2017.06.153 28709370

[B6] Aus Der Beek T. Weber F. A. Bergmann A. Hickmann S. Ebert I. Hein A. (2016). Pharmaceuticals in the environment—global occurrences and perspectives. *Environ. Toxicol. Chem.* 35 823–835. 10.1002/etc.3339 26666847

[B7] Balcázar J. L. Subirats J. Borrego C. M. (2015). The role of biofilms as environmental reservoirs of antibiotic resistance. *Front. Microbiol.* 6:1216. 10.3389/fmicb.2015.01216 26583011 PMC4628128

[B8] Bengtsson-Palme J. Kristiansson E. Larsson D. G. J. (2018). Environmental factors influencing the development and spread of antibiotic resistance. *FEMS Microbiol. Rev.* 42 68–80. 10.1093/femsre/fux053 29069382 PMC5812547

[B9] Besemer K. (2016). Biodiversity, community structure and function of biofilms in stream ecosystems. *Res. Microbiol.* 166 774–781. 10.1016/j.resmic.2015.05.006.Biodiversity PMC4601154 26027773

[B10] Bottery M. J. Pitchford J. W. Friman V.-P. (2020). Ecology and evolution of antimicrobial resistance in bacterial communities. *ISME J.* 15 939–948. 10.1038/s41396-020-00832-7 33219299 PMC8115348

[B11] Buelow E. Bayjanov J. R. Majoor E. Willems R. J. Bonten M. J. Schmitt H. (2018). Limited influence of hospital wastewater on the microbiome and resistome of wastewater in a community sewerage system. *FEMS Microbiol. Ecol.* 94:fiy087. 10.1093/femsec/fiy087 29767712

[B12] Buelow E. Rico A. Gaschet M. Lourenço J. Kennedy S. P. Wiest L. (2020). Hospital discharges in urban sanitation systems?: long-term monitoring of wastewater resistome and microbiota in relationship to their eco-exposome. *Water Res. X* 7:100045. 10.1016/j.wroa.2020.100045 32072151 PMC7013138

[B13] Cai X. Yao L. Sheng Q. Jiang L. Dahlgren R. A. Wang T. (2018). Properties of bacterial communities attached to artificial substrates in a hypereutrophic urban river. *AMB Express* 8:22. 10.1186/s13568-018-0545-z 29453676 PMC5815975

[B14] Cairns J. Ruokolainen L. Hultman J. Tamminen M. Virta M. Hiltunen T. (2018). Ecology determines how low antibiotic concentration impacts community composition and horizontal transfer of resistance genes. *Commun. Biol.* 1:35. 10.1038/s42003-018-0041-7 30271921 PMC6123812

[B15] Carles L. Wullschleger S. Joss A. Eggen R. I. L. Schirmer K. Schuwirth N. (2021). Impact of wastewater on the microbial diversity of periphyton and its tolerance to micropollutants in an engineered flow-through channel system. *Water Res.* 203:117486. 10.1016/J.WATRES.2021.117486 34412020

[B16] Carney R. L. Mitrovic S. M. Jeffries T. Westhorpe D. Curlevski N. Seymour J. R. (2015). River bacterioplankton community responses to a high inflow event. *Aquat. Microb. Ecol.* 75 187–205. 10.3354/ame01758

[B17] Caucci S. Karkman A. Cacace D. Rybicki M. Timpel P. Voolaid V. (2016). Seasonality of antibiotic prescriptions for outpatients and resistance genes in sewers and wastewater treatment plant outflow. *FEMS Microbiol. Ecol.* 92 1–10. 10.1093/femsec/fiw060 27073234

[B18] Chen J. Li W. Zhang J. Qi W. Li Y. Chen S. (2020). Prevalence of antibiotic resistance genes in drinking water and biofilms: the correlation with the microbial community and opportunistic pathogens. *Chemosphere* 259:127483. 10.1016/j.chemosphere.2020.127483 32634723

[B19] Chu B. T. T. Petrovich M. L. Chaudhary A. Wright D. Murphy B. Wells G. (2018). Metagenomics reveals the impact of wastewater treatment plants on the dispersal of microorganisms and genes in aquatic sediments. *Appl. Environ. Microbiol.* 84:e02168-17. 10.1128/AEM.02168-17 29269503 PMC5812944

[B20] Crabbé A. Jensen P. Ø Bjarnsholt T. Coenye T. (2019). Antimicrobial tolerance and metabolic adaptations in microbial biofilms. *Trends Microbiol.* 27 850–863. 10.1016/j.tim.2019.05.003 31178124

[B21] Danner M. C. Robertson A. Behrends V. Reiss J. (2019). Antibiotic pollution in surface fresh waters: occurrence and effects. *Sci. Total Environ.* 664 793–804. 10.1016/j.scitotenv.2019.01.406 30763859

[B22] Devarajan N. Laffite A. Graham N. D. Meijer M. Prabakar K. Mubedi J. I. (2015). Accumulation of clinically relevant antibiotic-resistance genes, bacterial load, and metals in freshwater lake sediments in central europe. *Environ. Sci. Technol.* 49 6528–6537. 10.1021/ACS.EST.5B01031 25933054

[B23] Drlica K. Zhao X. (2007). Mutant selection window hypothesis updated. *Clin. Infect. Dis.* 44 681–688. 10.1086/511642 17278059

[B24] Dunai A. Spohn R. Farkas Z. Lázár V. Györkei Á Apjok G. (2019). Rapid decline of bacterial drug-resistance in an antibiotic-free environment through phenotypic reversion. *eLife* 8:e47088. 10.7554/ELIFE.47088 31418687 PMC6707769

[B25] Elseviers M. M. Ferech M. Vander Stichele R. H. Goossens H., and Esac Project Group (2007). Antibiotic use in ambulatory care in Europe (ESAC data 1997 – 2002): trends, regional differences and seasonal fluctuations. *Pharmacoepidemiol. Drug Saf.* 16 115–123. 10.1002/pds.1244 16700079

[B26] Flemming H. Wingender J. Szewzyk U. Steinberg P. Rice S. A. Kjelleberg S. (2016). Biofilms?: an emergent form of bacterial life. *Nat. Rev. Microbiol.* 14 563–575. 10.1038/nrmicro.2016.94 27510863

[B27] Friman V. P. Guzman L. M. Reuman D. C. Bell T. (2015). Bacterial adaptation to sublethal antibiotic gradients can change the ecological properties of multitrophic microbial communities. *Proc. R. Soc. B Biol. Sci.* 282:20142920. 10.1098/rspb.2014.2920 25833854 PMC4426614

[B28] Gebreyohannes G. Nyerere A. Bii C. Sbhatu D. B. (2019). Challenges of intervention, treatment, and antibiotic resistance of biofilm-forming microorganisms. *Heliyon* 5:e02192. 10.1016/j.heliyon.2019.e02192 31463386 PMC6709409

[B29] Glibota N. Grande M. J. Galvez A. Ortega E. (2020). Genetic determinants for metal tolerance and antimicrobial resistance detected in bacteria isolated from soils of olive tree farms. *Antibiotics* 9:476. 10.3390/antibiotics9080476 32756388 PMC7459592

[B30] Gönder Z. B. Kara E. M. Çelik B. Ö Vergili I. Kaya Y. Altinkum S. M. (2021). Detailed characterization, antibiotic resistance and seasonal variation of hospital wastewater. *Environ. Sci. Pollut. Res.* 28 16380–16393. 10.1007/s11356-020-12221-w 33387316

[B31] González-Plaza J. J. Blau K. Milaković M. Jurina T. Smalla K. Udiković-Kolić N. (2019). Antibiotic-manufacturing sites are hot-spots for the release and spread of antibiotic resistance genes and mobile genetic elements in receiving aquatic environments. *Environ. Int.* 130:104735. 10.1016/J.ENVINT.2019.04.007 31260930

[B32] Gothwal R. Shashidhar T. (2015). Antibiotic pollution in the environment: a review. *Clean Soil Air Water* 43 479–489. 10.1002/clen.201300989

[B33] Gullberg E. (2014). *Selection of Resistance at Very low Antibiotic Concentrations.* Uppsala: University of Uppsala.

[B34] Gullberg E. Cao S. Berg O. G. Ilbäck C. Sandegren L. Hughes D. (2011). Selection of resistant bacteria at very low antibiotic concentrations. *PLoS Pathog.* 7:e1002158. 10.1371/journal.ppat.1002158 21811410 PMC3141051

[B35] Harnisz M. Kiedrzyńska E. Kiedrzyński M. Korzeniewska E. Czatzkowska M. Koniuszewska I. (2020). The impact of WWTP size and sampling season on the prevalence of antibiotic resistance genes in wastewater and the river system. *Sci. Total Environ.* 741:140466. 10.1016/j.scitotenv.2020.140466 32886993

[B36] Hathroubi S. Mekni M. A. Domenico P. Nguyen D. Jacques M. (2017). Biofilms: microbial shelters against antibiotics. *Microb. Drug Resist.* 23 147–156. 10.1089/mdr.2016.0087 27214143

[B37] Hektoen H. Berge J. A. Hormazábal V. Yndestad M. (1995). Persistence of antibacterial agents in marine sediments. *Aquaculture* 133 175–184. 10.1016/0044-8486(94)00310-K

[B38] Herlemann D. P. R. Labrenz M. Jürgens K. Bertilsson S. Waniek J. J. Andersson A. F. (2011). Transitions in bacterial communities along the 2000?km salinity gradient of the Baltic Sea. *ISME J.* 5 1571–1579. 10.1038/ismej.2011.41 21472016 PMC3176514

[B39] Kaevska M. Videnska P. Sedlar K. Slana I. (2016). Seasonal changes in microbial community composition in river water studied using 454-pyrosequencing. *SpringerPlus* 5:409. 10.1186/S40064-016-2043-6 27069829 PMC4821842

[B40] Khan S. Beattie T. K. Knapp C. W. (2017). The use of minimum selectable concentrations (MSCs) for determining the selection of antimicrobial resistant bacteria. *Ecotoxicology* 26 283–292. 10.1007/s10646-017-1762-y 28155034 PMC5318476

[B41] Khatoon Z. McTiernan C. D. Suuronen E. J. Mah T. F. Alarcon E. I. (2018). Bacterial biofilm formation on implantable devices and approaches to its treatment and prevention. *Heliyon* 4:e01067. 10.1016/j.heliyon.2018.e01067 30619958 PMC6312881

[B42] Kim M.-K. Zoh K.-D. (2016). Occurrence and removals of micropollutants in water environment. *Environ. Eng. Res.* 21 319–332. 10.4491/EER.2016.115

[B43] Köchling T. Sanz J. L. Galdino L. Florencio L. Kato M. T. (2017). Impact of pollution on the microbial diversity of a tropical river in an urbanized region of northeastern Brazil. *Int. Microbiol.* 20 11–24. 10.2436/20.1501.01.281 28581018

[B44] Kunhikannan S. Thomas C. J. Franks A. E. Mahadevaiah S. Kumar S. Petrovski S. (2021). Environmental hotspots for antibiotic resistance genes. *MicrobiologyOpen* 10:e1197. 10.1002/MBO3.1197 34180594 PMC8123917

[B45] Larsson D. G. J. Andremont A. Bengtsson-Palme J. Brandt K. K. de Roda Husman A. M. Fagerstedt P. (2018). Critical knowledge gaps and research needs related to the environmental dimensions of antibiotic resistance. *Environ. Int.* 117 132–138. 10.1016/j.envint.2018.04.041 29747082

[B46] Li Q. Zhang Q. (2020). Prevalence and pollution characteristics of antibiotic resistant genes in one high anthropogenically-impacted river. *PLoS One* 15:e0231128. 10.1371/JOURNAL.PONE.0231128 32271821 PMC7145097

[B47] Li W. Shi Y. Gao L. Liu J. Cai Y. (2012). Occurrence of antibiotics in water, sediments, aquatic plants, and animals from Baiyangdian Lake in North China. *Chemosphere* 89 1307–1315. 10.1016/j.chemosphere.2012.05.079 22698376

[B48] Liang D. W. Fang H. H. P. Zhang T. (2009). Microbial characterization and quantification of an anaerobic sludge degrading dimethyl phthalate. *J. Appl. Microbiol.* 106 296–305. 10.1111/j.1365-2672.2008.04003.x 19120614

[B49] Lopatkin A. J. Meredith H. R. Srimani J. K. Pfeiffer C. Durrett R. You L. (2017). Persistence and reversal of plasmid-mediated antibiotic resistance. *Nat. Commun.* 8:1689. 10.1038/s41467-017-01532-1 29162798 PMC5698434

[B50] Luo Y. Guo W. Ngo H. H. Nghiem L. D. Hai F. I. Zhang J. (2014). A review on the occurrence of micropollutants in the aquatic environment and their fate and removal during wastewater treatment. *Sci. Total Environ.* 473–474 619–641. 10.1016/J.SCITOTENV.2013.12.065 24394371

[B51] Mao G. Liang J. Wang Q. Zhao C. Bai Y. Liu R. (2021). Epilithic biofilm as a reservoir for functional virulence factors in wastewater-dominant rivers after WWTP upgrade. *J. Environ. Sci. (China)* 101 27–35. 10.1016/j.jes.2020.05.014 33334522

[B52] McCarty P. L. (2018). What is the best biological process for nitrogen removal: when and why?. *Environ. Sci. Technol.* 52 3835–3841. 10.1021/acs.est.7b05832 29510030

[B53] Moon C. D. Young W. Maclean P. H. Cookson A. L. Bermingham E. N. (2018). Metagenomic insights into the roles of *Proteobacteria* in the gastrointestinal microbiomes of healthy dogs and cats. *MicrobiologyOpen* 7 1–20. 10.1002/mbo3.677 29911322 PMC6182564

[B54] Murray A. K. Stanton I. C. Wright J. Zhang L. Snape J. Gaze W. H. (2020). The “selection end points in communities of bacteria” (Select) method: a novel experimental assay to facilitate risk assessment of selection for antimicrobial resistance in the environment. *Environ. Health Perspect.* 128:107007. 10.1289/EHP6635 33084388 PMC7577113

[B55] Muziasari W. I. Managaki S. Pärnänen K. Karkman A. Lyra C. Tamminen M. (2014). Sulphonamide and trimethoprim resistance genes persist in sediments at baltic sea aquaculture farms but are not detected in the surrounding environment. *PLoS One* 9:e92702. 10.1371/JOURNAL.PONE.0092702 24651770 PMC3961581

[B56] Pal C. Asiani K. Arya S. Rensing C. Stekel D. J. Larsson D. G. J. (2017). Metal resistance and its association with antibiotic resistance. *Adv. Microb. Physiol.* 70 261–313. 10.1016/bs.ampbs.2017.02.001 28528649

[B57] Partridge S. R. Kwong S. M. Firth N. Jensen S. O. (2018). Mobile genetic elements associated with antimicrobial resistance. *Clin. Microbiol. Rev.* 31:e00088-17. 10.1128/CMR.00088-17 30068738 PMC6148190

[B58] Patel M. Kumar R. Kishor K. Mlsna T. Pittman C. U. Mohan D. (2019). Pharmaceuticals of emerging concern in aquatic systems: chemistry, occurrence, effects, and removal methods. *Chem. Rev.* 119 3510–3673. 10.1021/acs.chemrev.8b00299 30830758

[B59] Proia L. von Schiller D. Sànchez-Melsió A. Sabater S. Borrego C. M. Rodríguez-Mozaz S. (2016). Occurrence and persistence of antibiotic resistance genes in river biofilms after wastewater inputs in small rivers. *Environ. Pollut.* 210 121–128. 10.1016/J.ENVPOL.2015.11.035 26708766

[B60] Singer A. C. Shaw H. Rhodes V. Hart A. (2016). Review of antimicrobial resistance in the environment and its relevance to environmental regulators. *Front. Microbiol.* 7:1728. 10.3389/fmicb.2016.01728 27847505 PMC5088501

[B61] Stalder T. Barraud O. Jové T. Casellas M. Gaschet M. Dagot C. (2014). Quantitative and qualitative impact of hospital effluent on dissemination of the integron pool. *ISME J.* 8 768–777. 10.1038/ismej.2013.189 24152716 PMC3960533

[B62] Stanton I. C. Bethel A. Leonard A. F. C. Gaze W. H. Garside R. (2020). What is the research evidence for antibiotic resistance exposure and transmission to humans from the environment? A systematic map protocol. *Environ. Evid.* 9:12. 10.1186/s13750-020-00197-6 32518638 PMC7268584

[B63] Suda K. J. Hicks L. A. Roberts R. M. Hunkler R. J. Taylor T. H. (2014). Trends and seasonal variation in outpatient antibiotic prescription rates in the United States, 2006 to 2010. *Antimicrob. Agents Chemother.* 58:2763. 10.1128/AAC.02239-13 24590486 PMC3993241

[B64] Tian Z. Palomo A. Zhang H. Luan X. Liu R. Awad M. (2020). Minimum influent concentrations of oxytetracycline, streptomycin and spiramycin in selecting antibiotic resistance in biofilm type wastewater treatment systems. *Sci. Total Environ.* 720:137531. 10.1016/j.scitotenv.2020.137531 32325576

[B65] Valdés M. E. Lucia H. Carolina R. C. Adonis G. Damià B. Sara R. (2021). Distribution of antibiotics in water, sediments and biofilm in an urban river (Córdoba, Argentina, LA). *Environ. Pollut.* 269:116133. 10.1016/J.ENVPOL.2020.116133 33316497

[B66] Wales A. D. Davies R. H. (2015). Co-selection of resistance to antibiotics, biocides and heavy metals, and its relevance to foodborne pathogens. *Antibiotics* 4:567. 10.3390/ANTIBIOTICS4040567 27025641 PMC4790313

[B67] Wang J. Fan H. He X. Zhang F. Xiao J. Yan Z. (2021). Response of bacterial communities to variation in water quality and physicochemical conditions in a river-reservoir system. *Glob. Ecol. Conserv.* 27:e01541. 10.1016/J.GECCO.2021.E01541

[B68] Woegerbauer M. Bellanger X. Merlin C. (2020). Cell-free DNA: an underestimated source of antibiotic resistance gene dissemination at the interface between human activities and downstream environments in the context of wastewater reuse. *Front. Microbiol.* 11:671. 10.3389/fmicb.2020.00671 32390973 PMC7192050

[B69] Yang Q. E. Agouri S. R. Tyrrell J. M. Walsh T. R. (2018). Heavy metal resistance genes are associated with bla NDM-1 - and bla CTX-M-15 -carrying *Enterobacteriaceae*. *Antimicrob. Agents Chemother.* 62:e02642-17. 10.1128/AAC.02642-17 29507071 PMC5923091

[B70] Zhang H. Du M. Jiang H. Zhang D. Lin L. Ye H. (2015). Occurrence, seasonal variation and removal efficiency of antibiotics and their metabolites in wastewater treatment plants, Jiulongjiang River Basin, South China. *Environ. Sci. Proc. Impacts* 17 225–234. 10.1039/c4em00457d 25503570

[B71] Zhang L. Shen Z. Fang W. Gao G. (2019). Composition of bacterial communities in municipal wastewater treatment plant. *Sci. Total Environ.* 689 1181–1191. 10.1016/j.scitotenv.2019.06.432 31466158

[B72] Zhao R. Feng J. Yin X. Jie L. Wenjie F. Berendonk T. U. (2018). Antibiotic resistome in landfill leachate from different cities of China deciphered by metagenomic analysis. *Water Res.* 134 126–139. 10.1016/j.watres.2018.01.063 29407646

[B73] Zhuang M. Achmon Y. Cao Y. Liang X. Chen L. Wang H. (2021). Distribution of antibiotic resistance genes in the environment. *Environ. Pollut.* 285:117402. 10.1016/j.envpol.2021.117402 34051569

