# Supplementary material for: River Biofilms Microbiome and Resistome Responses to Wastewater Treatment Plant Effluents Containing Antibiotics
Source: Front Microbiol. 2022 Feb 9;13:795206. doi: 10.3389/fmicb.2022.795206 (PMC8863943; doi:10.3389/fmicb.2022.795206)
Supplement: Supplementary file 1 [file Presentation_1.zip › Supplementary Figures 1-9 and Tables 1-4.DOCX]

Supplementary Material

River biofilms microbiome and resistome responses to wastewater treatment plant effluents containing antibiotics

**Olha MATVIICHUK^1,2^, Leslie MONDAMERT^1^, Claude GEFFROY^1^, Margaux GASCHET^2^, Christophe DAGOT^2^, Jérôme LABANOWSKI^1*^**

^1^Institut de Chimie des Milieux et Matériaux de Poitiers, UMR CNRS 7285, University of Poitiers, France.

^2^UMR INSERM 1092, Limoges, France.

*** Correspondence:**Jérôme Labanowski
jerome.labanowski@univ-poitiers.fr


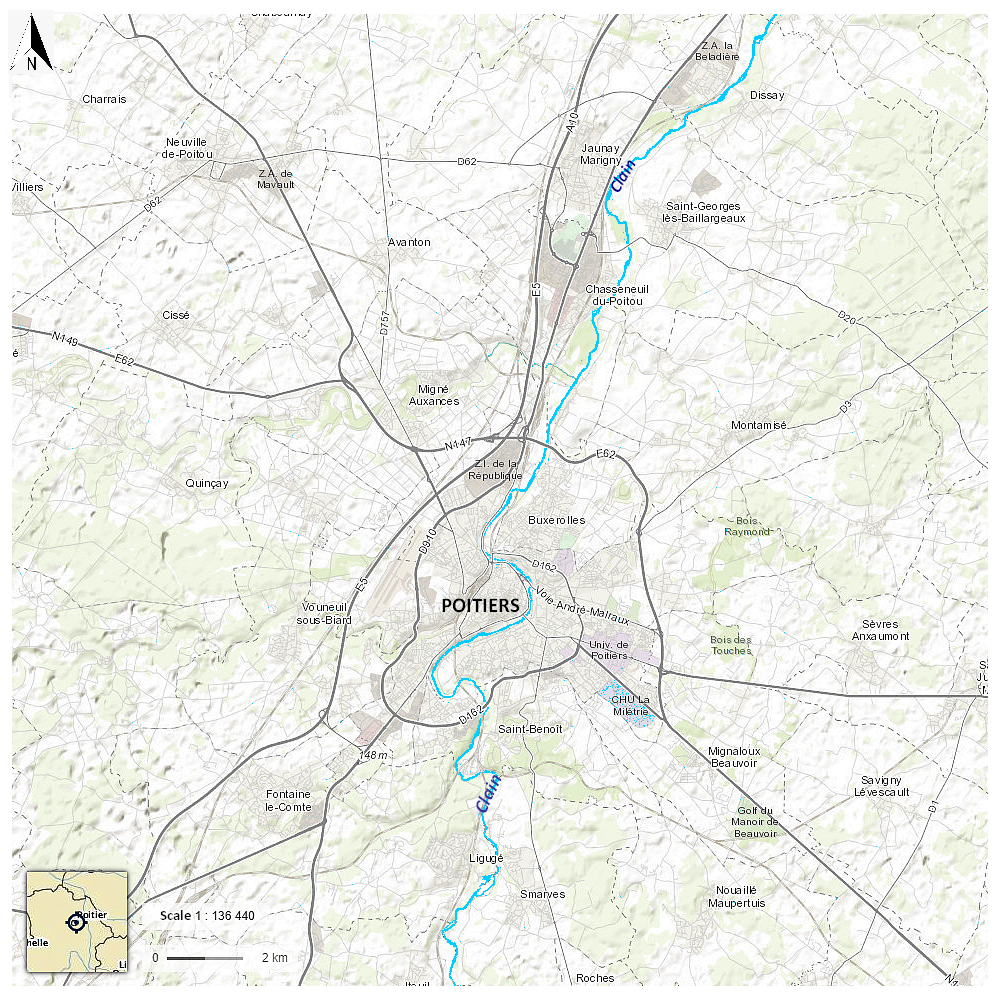

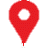

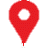

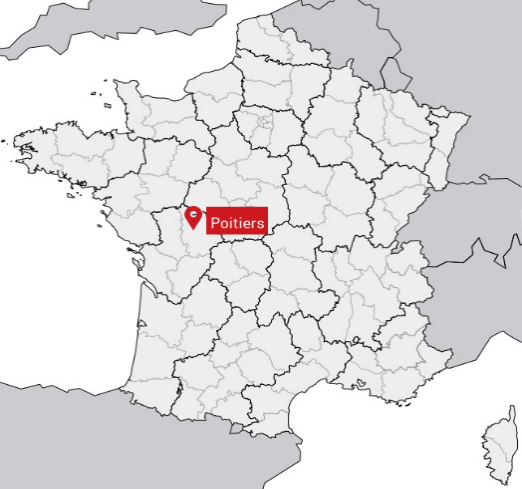


**Paris**


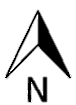


**WWTP**

**UPS**

**DWS**


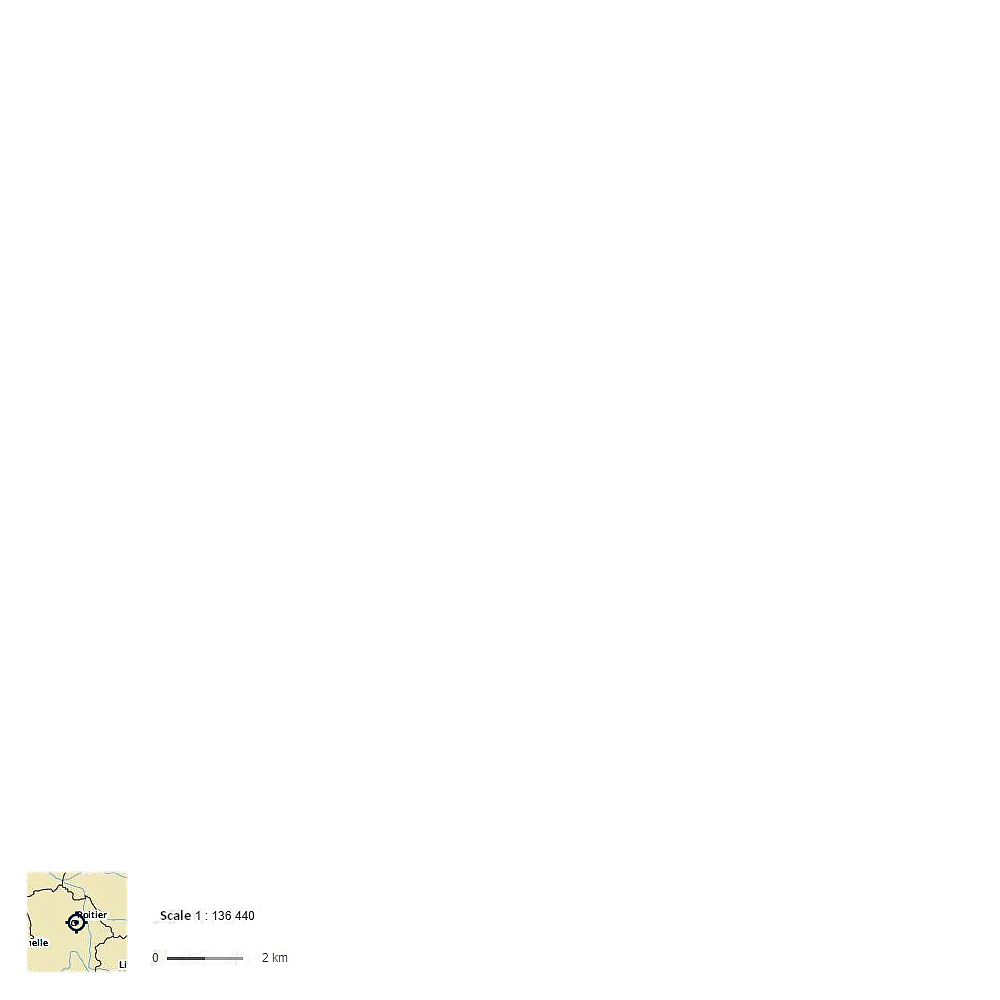


**Figure S1.** Sketch map showing the location of sampling sites (one upstream (UPS) and second downstream (DWS) from the wastewater treatment plant (WWTP)) in the Clain river.

The Poitiers’ wastewater treatment plant (La Folie) is capable of treating wastewater produced by 160,000 population equivalents. The WWTP has two identical and parallel treatment lines which ensure the elimination of carbon, nitrogen and phosphorus pollution in compliance with French regulations. The treatment includes the following stages:

Cycle 1: When wastewater arrives, it goes directly to pre-treatment, where it is screened, sand removed and degreased.

Cycle 2: biological treatment: After the removal of the above-mentioned wastes, the water undergoes biological treatment. This process consists in using bacteria to eliminate carbon, nitrogen, and phosphorus pollution. All these bacteria multiply and form sludge.

Cycle 3: water clarification: At the end of the biological treatment, the water is clarified by decantation. The clarified water is routed to a metering channel, which allows its flow rate to be determined.

The treated water is then discharged into the Clain river without disinfection. In France, no disinfection of urban wastewater treatment plant effluent is performed (or regulated). Some rare wastewater treatment plants use ozone, but to decompose/oxidize organic pollutants (pharmaceuticals, etc.). Chlorination of treated wastewater is never used for discharge into the aquatic environment because of the risk of formation of disinfection by-products (THM, AOX).

**
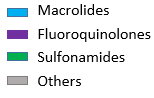

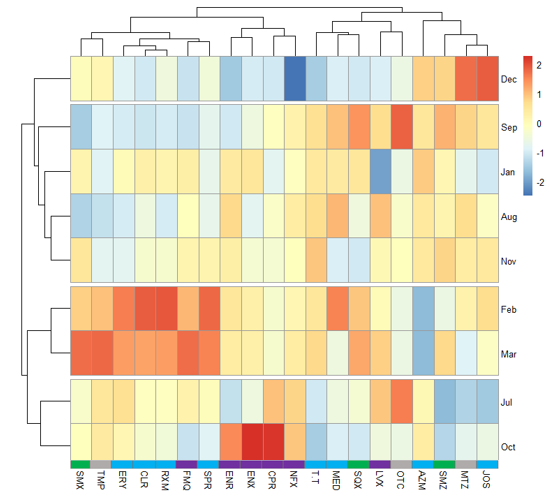

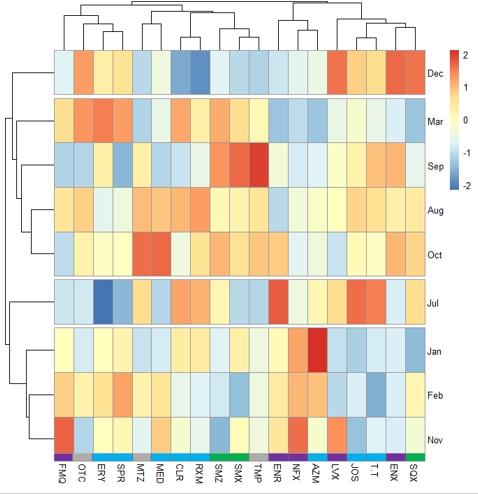
A** **B**

Figure S2. Cluster heatmaps of antibiotic concentration dynamics in river biofilms. (A) - UPS and (B) - DWS of the WWTP*.*

*
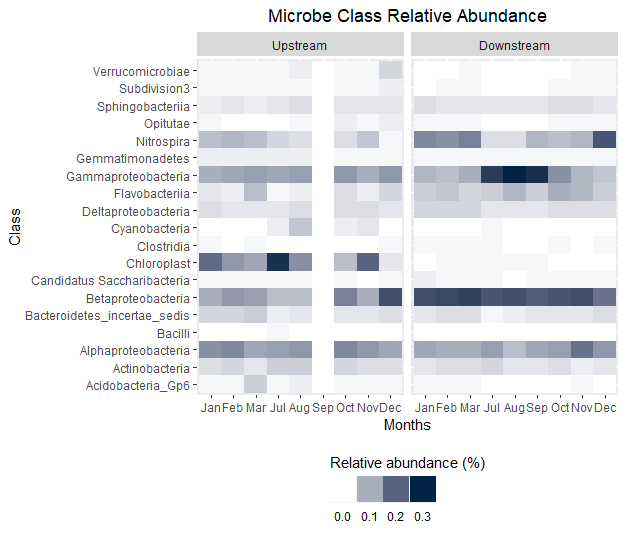
*Figure S3. Relative abundance of bacteria abundance at class level identified in metagenomes isolated from biofilms collected UPS and DWS of the WWTP*.* Results for September (UPS) are not included due to the problem during the analysis.

| Concomitant diseases | References |
| --- | --- |
| Dental caries (tooth decay) | (Li, Wyllie and Jensen, 2021) |
| Urinary, wound, eye and respiratory infections, endocarditis, osteomyelitis, septicemia, and meningitis | (Buckle, 2015) |
| Infection in the urinary, blood and gastrointestinal tracts, respiratory system, dermis, soft tissue, bacteremia | (Wu and Li, 2014) |
| Diarrhea, sepsis, keratitis, meningitis, pneumonia and osteomyelitis | (Chen *et al.*, 2013; Galloway and Cohen, 2021) |
| Pulmonary disease (tuberculosis), Hansen's disease (leprosy) | (Payeur, 2014) |
| Pontiac fever, pneumonia | (Diederen, 2008) |
| Bacteremia, affecting immunocompromised individuals | (Smith, 2014) |
| Pneumonia, skin, blood infections, meningitis, epiglotittis | (Geme and Rempe, 2018) |
| Intestinal infection (shigellosis), diarrhea | (Drancourt, 2010; Bennish and Ahmed, 2020) |
| Infections in the intestinal tract, skin, urinary tract, blood, heart | (Jha, Bais and Vivanco, 2005) |
| Botulism, tetanus, neonatal sepsis, and enteric infections | (Qureshi and Qamar, 2020) |
| Infections of the urinary tract, respiratory tract, blood, neonatal meningitis and brain abscess, gastroenteritis | (Antonara and Ardura, 2018) |
| Pneumonia, urinary tract infections, septic arthritis, and melioidosis | (Wanger *et al.*, 2017) |
| Pertussis (whooping cough) | (Soumana, Linz and Harvill, 2017) |
| Abscesses, anthrax, ear, respiratory and urinary tract infections, endocarditis, meningitis, ophthalmitis, osteomyelitis, peritonitis | (Turnbull, 1996; Bottone, 2010) |
| Gastrointestinal and urinary tract infections | (Drancourt, 2010; Janda and Abbott, 2010) |
| Infections in the blood, urinary tract, and lungs (pneumonia) | (Wong *et al.*, 2017) |


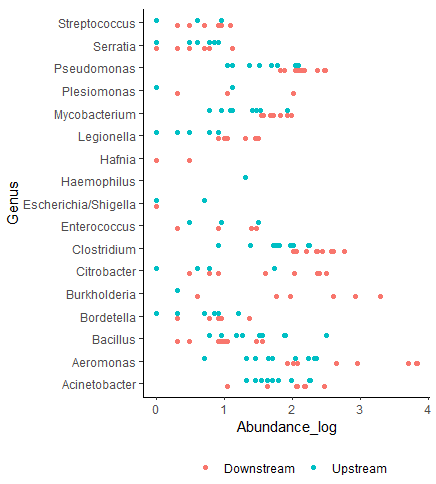


**Figure S4.** Relative abundance of potential pathogenic genera in collected biofilms and their related diseases.


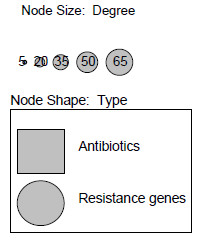

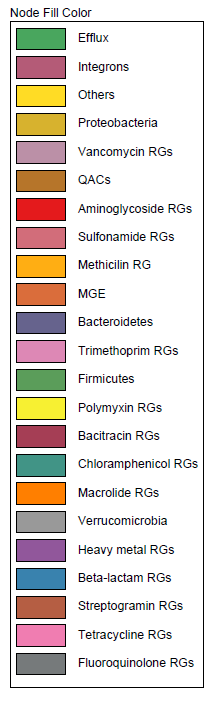


**A**

**B**


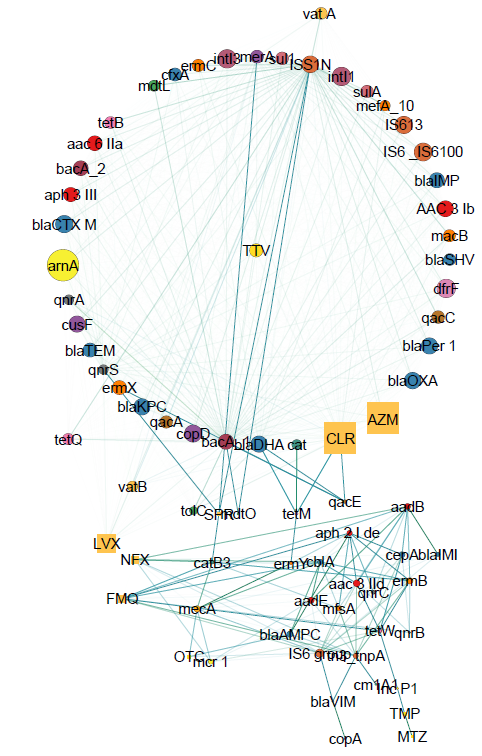

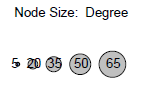

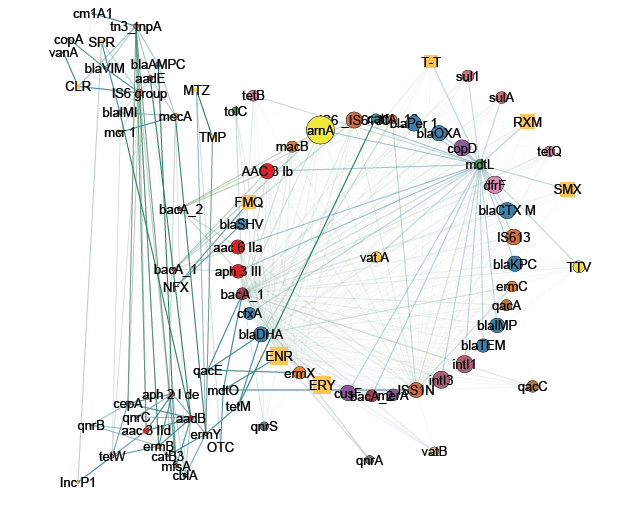


**Figure S5.** Network analysis revealing co-occurrence patterns between resistome and ATBs at concentration (A) <MSC, (B) between MSC-MIC UPS of the WWTP.


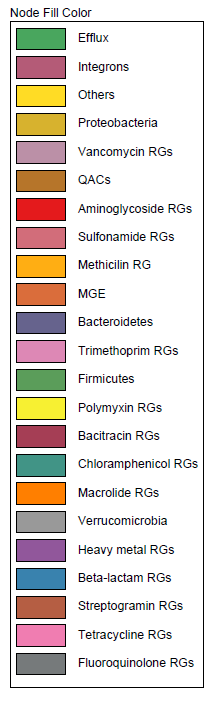


**B**

**A**


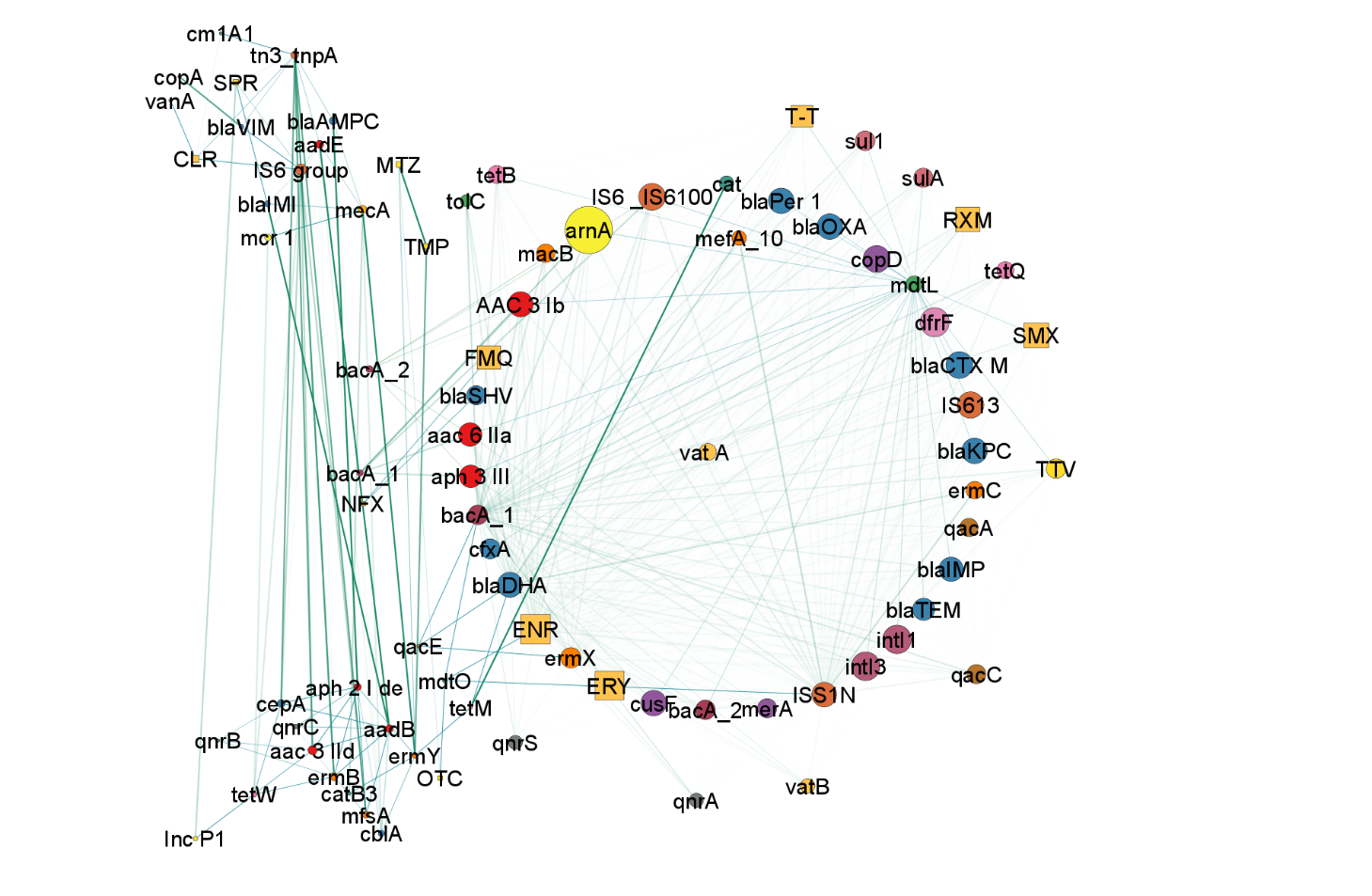

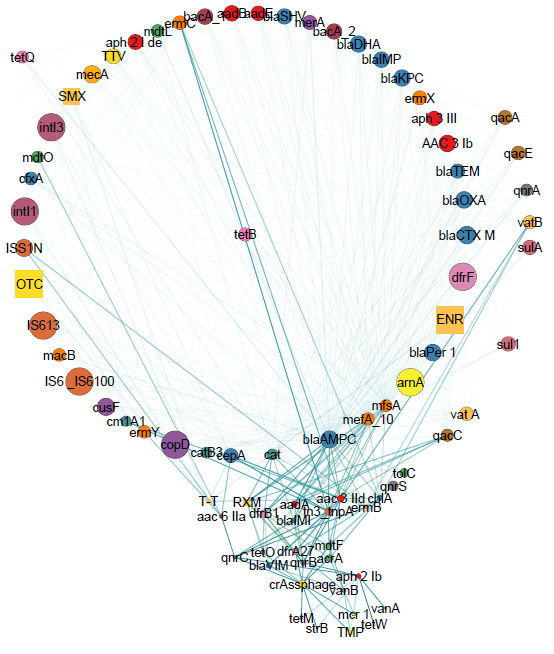

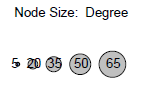

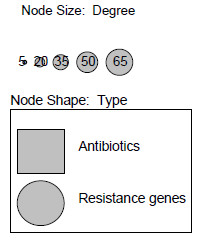


**Figure S6.** Network analysis revealing co-occurrence patterns between resistome and ATBs at concentration (A) >MIC UPS of the WWTP, (B) < MSC DWS of the WWTP.


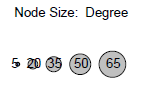

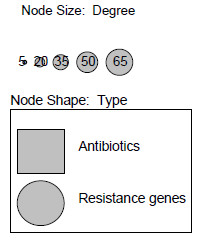

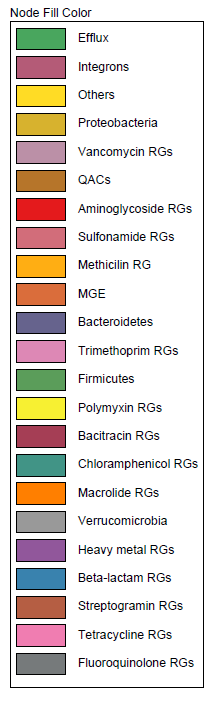

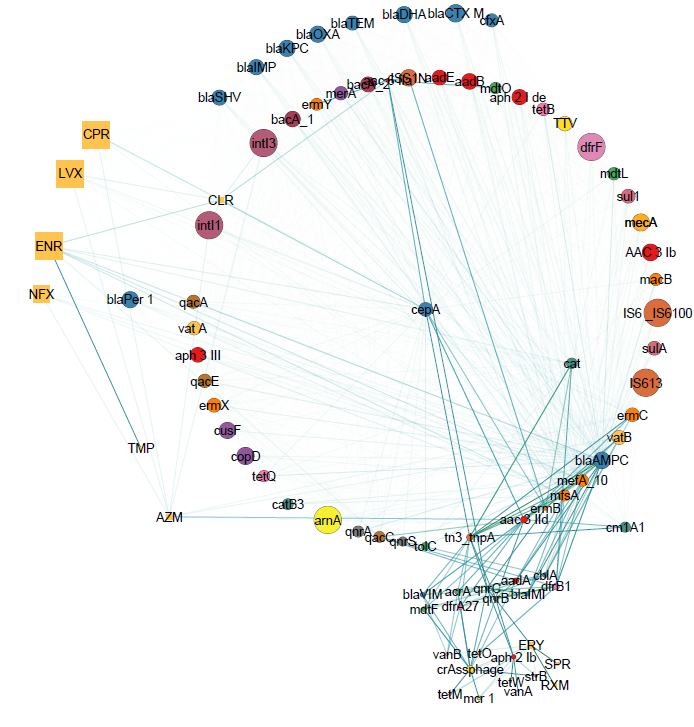

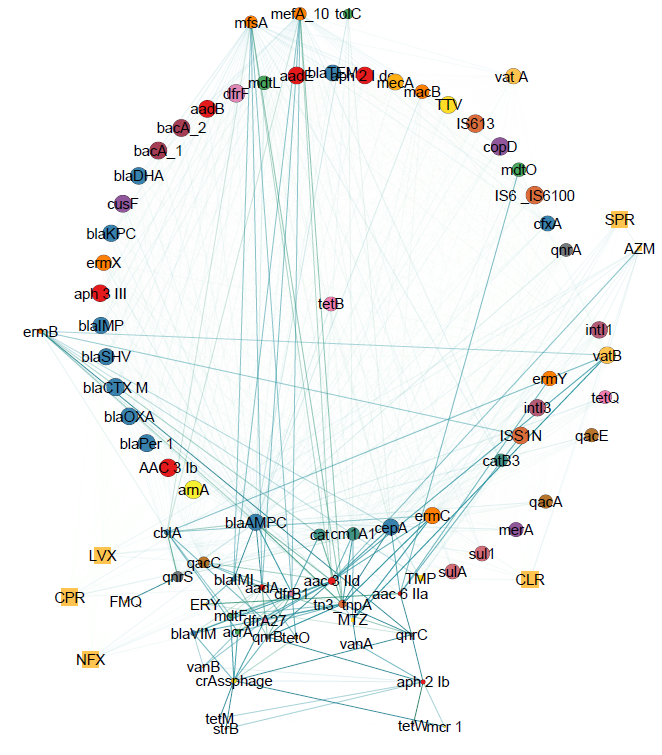


**A**

**B**

**Figure S7.** Network analysis revealing co-occurrence patterns between resistome and ATBs at concentration (A) between MSC-MIC, (B) >MIC DWS of the WWTP.


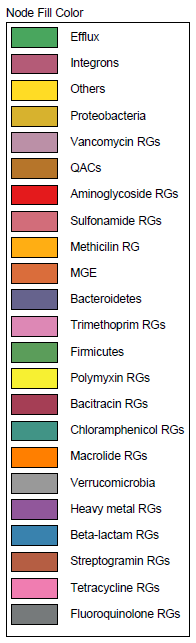

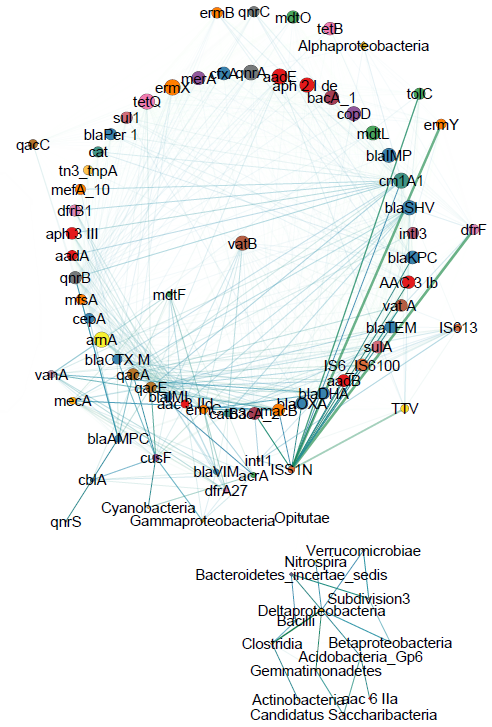


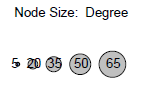

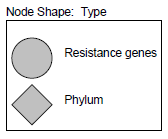

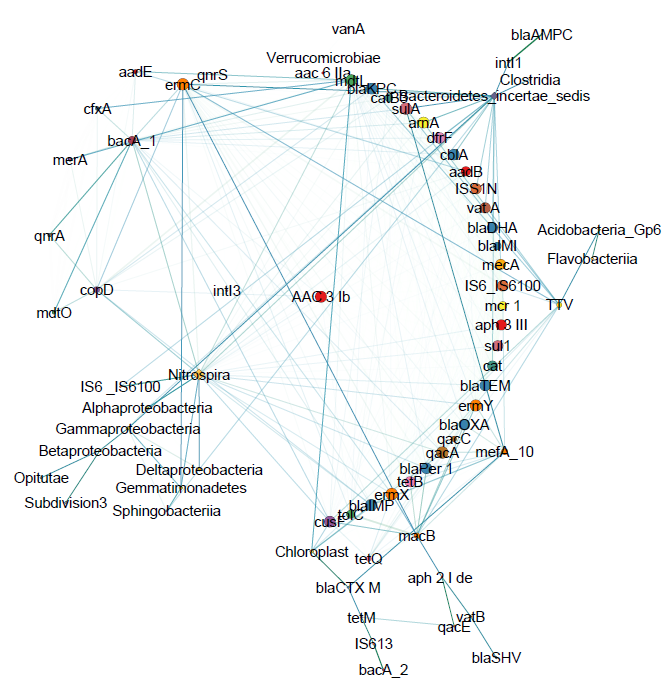


**B**

**A**

**Figure S8.** Network analysis revealing co-occurrence patterns between resistome and microbiome (A) UPS, (B) DWS of the WWTP.

**Figure S9. Absolute copy number of 16S rRNA genes in biofilms collected upstream (UPS) and downstream (DWS) of the WWTP of Poitiers determined by the standard qPCR.**

**Table S1.** Chemical properties of selected antibiotics.

| **Antibiotic** | Enrofloxacin (ENR) | Levofloxacin (LVX) | Norfloxacin (NFX) |
| --- | --- | --- | --- |
| **Molecular weight (g/mol)**  **CAS No**  **pKa**  **Log P**  **Chemical structure** | 359.401  93106-60-6  5.88-8.77  1.15*  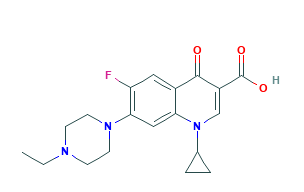 | 361.373  100986-85-4  5.45-6.2  2.1  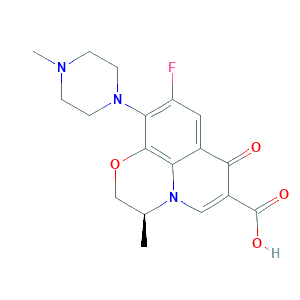 | 319.336  70458-96-7  5.77- 8.68  -1.03  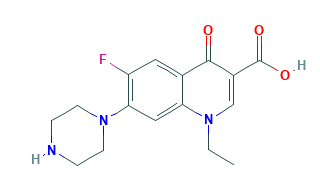 |
| Ciprofloxacin (CPR) | Flumequine (FMQ) | Enoxacin (ENX) | Erythromycin (ERY) |
| 331.347  85721-33-1  6.09  0.28  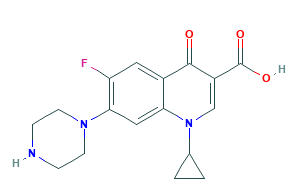 | 261.252  42835-25-6  -4.3-6.0  1.6  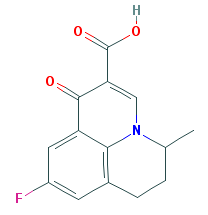 | 320.324  74011-58-8  5.5-8.59  -0.2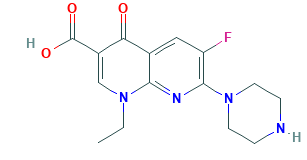 | 733.937  114-07-8  8.88 (at 25 °C)  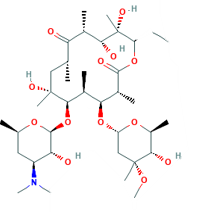 3.06 |
| Roxithromycin (RXM) | Clarithromycin (CLR) | Tylosin tartrate (T-T) | Midecamycin (MED) |
| 837.058  80214-83-1  9.08-12.45  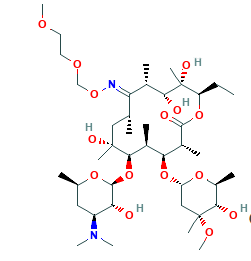1.7 | 747.964  81103-11-9  8.99 (at 25 °C)  3.16  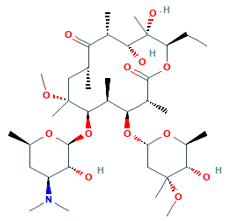 | 1066.198  74610-55-2  - 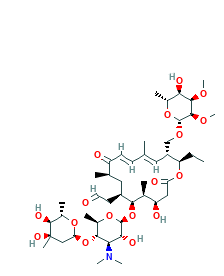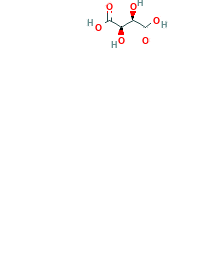 | 813.968  35457-80-8  6.9  2.22  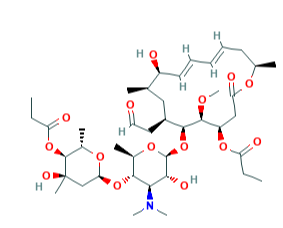 |
| Spiramycin (SPR) | Jocamycin (JOS) | Azithromycin (AZM) | Oxytetracycline (OTC) |
| 843.053  8025-81-8  7.88  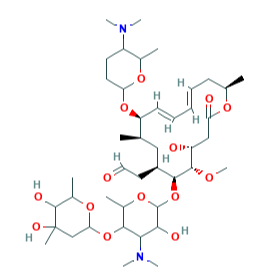1.87 | 827.995  16846-24-5  7.9*  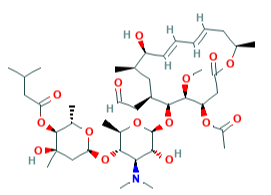3.22* | 748.984  83905-01-5  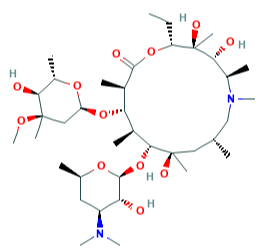8.5  4.02 | 460.439  79-57-2  3.27 (at 25 °C)  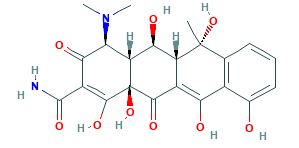-0.90 |
| Trimethoprim (TMP) | Metronidazole (MTZ) | Sulfamethazine (SMZ) | Sulfamethoxazole (SMX) |
| 290.323  738-70-5  7.12 (at 20 °C)  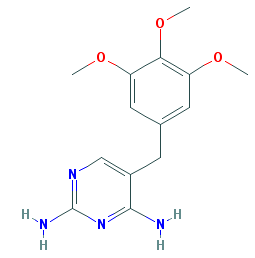 0.91 | 171.156  443-48-1  2.38  -0.02  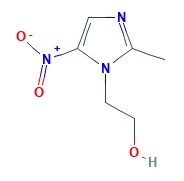 | 278.33  57-68-1  7.59  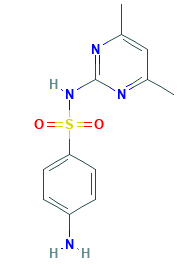0.89 | 253.276  723-46-6  1.6-5.8  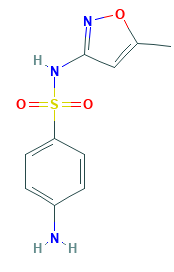 0.89 |
| Sulfaquinoxaline (SQX) |  |  |  |
| 300.336  59-40-5  5.1  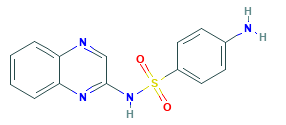1.68 |  |  |  |

**Table S2. Unidimensional test of equality of the means of the classes showing which variables (concentration of ATBs) are significantly different between studied sites.**

| Variable | Lambda | F | p-value |
| --- | --- | --- | --- |
| CPR | 0.498 | 16.120 | 0.001 |
| ENX | 0.892 | 1.947 | 0.182 |
| ENR | 0.538 | 13.718 | 0.002 |
| FMQ | 0.991 | 0.142 | 0.711 |
| LVX | 0.172 | 76.932 | < 0,0001 |
| NFX | 0.489 | 16.705 | 0.001 |
| AZM | 0.536 | 13.831 | 0.002 |
| CLR | 0.689 | 7.229 | 0.016 |
| ERY | 0.718 | 6.282 | 0.023 |
| JOS | 1.000 | 0.002 | 0.963 |
| MED | 1.000 | 0.000 | 0.991 |
| RXM | 0.716 | 6.361 | 0.023 |
| SPR | 0.472 | 17.898 | 0.001 |
| T-T | 0.780 | 4.505 | 0.050 |
| SMZ | 0.918 | 1.437 | 0.248 |
| SMX | 0.248 | 48.548 | < 0,0001 |
| SQX | 0.970 | 0.503 | 0.488 |
| MTZ | 0.960 | 0.667 | 0.426 |
| OTC | 0.541 | 13.556 | 0.002 |
| TMP | 0.921 | 1.378 | 0.258 |

**Table S3. Unidimensional test of equality of the means of the classes showing which variables (normalized abundance of resistance genes and MGEs) are significantly different between studied sites.**

| Variable | Lambda | F | p-value | Variable | Lambda | F | p-value |
| --- | --- | --- | --- | --- | --- | --- | --- |
| catB3 | 0.707 | 6.205 | 0.025 | mdtO | 0.864 | 2.368 | 0.145 |
| cat | 0.870 | 2.237 | 0.155 | tolC | 0.810 | 3.517 | 0.080 |
| cm1A1 | 0.717 | 5.926 | 0.028 | mdtL | 0.790 | 3.988 | 0.064 |
| aadE | 0.655 | 7.886 | 0.013 | qnrA | 0.795 | 3.856 | 0.068 |
| aac(6')-IIa | 0.990 | 0.155 | 0.699 | qnrB | 0.839 | 2.875 | 0.111 |
| aph(3')-III | 0.799 | 3.762 | 0.071 | qnrS | 0.684 | 6.926 | 0.019 |
| aph(2")-I(de) | 0.822 | 3.251 | 0.091 | qnrC | 0.906 | 1.563 | 0.230 |
| aadB | 0.659 | 7.778 | 0.014 | cusF | 0.722 | 5.772 | 0.030 |
| AAC(3)-Ib | 0.751 | 4.978 | 0.041 | copD | 0.760 | 4.744 | 0.046 |
| bacA_2 | 0.783 | 4.157 | 0.060 | merA | 0.863 | 2.386 | 0.143 |
| bacA_1 | 0.821 | 3.280 | 0.090 | qacA | 0.627 | 8.931 | 0.009 |
| cblA | 0.793 | 3.914 | 0.067 | qacC | 0.868 | 2.273 | 0.152 |
| cfxA | 0.848 | 2.693 | 0.122 | qacE | 0.664 | 7.592 | 0.015 |
| blaTEM | 0.739 | 5.291 | 0.036 | vanA | 0.818 | 3.338 | 0.088 |
| blaIMP | 0.803 | 3.671 | 0.075 | tetQ | 0.667 | 7.504 | 0.015 |
| blaAMPC | 0.749 | 5.021 | 0.041 | tetB | 0.839 | 2.888 | 0.110 |
| blaDHA | 0.695 | 6.589 | 0.021 | arnA | 0.733 | 5.475 | 0.034 |
| blaSHV | 0.679 | 7.083 | 0.018 | sul1 | 0.870 | 2.247 | 0.155 |
| blaKPC | 0.729 | 5.569 | 0.032 | sulA | 0.640 | 8.436 | 0.011 |
| blaIMI | 0.822 | 3.245 | 0.092 | mecA | 0.738 | 5.330 | 0.036 |
| blaOXA | 0.741 | 5.254 | 0.037 | dfrF | 0.744 | 5.158 | 0.038 |
| blaCTX-M | 0.757 | 4.814 | 0.044 | vatB | 0.622 | 9.115 | 0.009 |
| blaPer-1 | 0.808 | 3.563 | 0.079 | vat(A) | 0.699 | 6.451 | 0.023 |
| ermC | 0.720 | 5.841 | 0.029 | ISS1N | 0.789 | 4.001 | 0.064 |
| ermY | 0.773 | 4.400 | 0.053 | IS6 _IS6100 | 0.718 | 5.903 | 0.028 |
| mefA_10 | 0.832 | 3.032 | 0.102 | IS613 | 0.735 | 5.408 | 0.034 |
| macB | 0.667 | 7.492 | 0.015 | intI1 | 0.525 | 13.555 | 0.002 |
| ermX | 0.749 | 5.037 | 0.040 | intI3 | 0.567 | 11.468 | 0.004 |
|  |  |  |  | TTV | 0.806 | 3.611 | 0.077 |

**Table S4. Summary of minimum selective concentration (MSC) and minimum inhibitory concentration (MIC) of antibiotics used in this study as levels defining potentially favorable ranges for resistance selection published by (Bengtsson-Palme and Larsson, 2016) versus predicted no-effect concentrations for resistance published by (Murray *et al.*, 2020). Concentrations expressed in µg/L.**

| ATB | MSC ^a^ | MIC ^a^ | PNEC^R a^ | PNEC^R^ qPCR ^b^ | PNEC^R^ SELECT ^b^ |
| --- | --- | --- | --- | --- | --- |
| CPR | 1 | 2 | 0.06 | 0.78 | 0.05 |
| AZM | 4 | 16 | 0.25 | 50 | 50 |
| CLR | 2 | 8 | 0.25 | 50 | 250 |
| ERY | 8 | 16 | 1 | 50 | 1250 |
| TMP | 8 | 16 | 0.5 | 3.125 | 1.56 |

^a^ – values given by Bengtsson-Palme and Larsson (2016) calculated based on data obtained from pure cultures; ^b^ – values given by Murray *et al.* (2020) obtained based on data collected from multispecies communities.

Antonara, S. and Ardura, M. I. (2018) ‘Citrobacter Species’, *Principles and Practice of Pediatric Infectious Diseases*. Elsevier, pp. 827-829.e1. doi: 10.1016/B978-0-323-40181-4.00141-9.

Bengtsson-Palme, J. and Larsson, J. (2016) ‘Concentrations of antibiotics predicted to select for resistant bacteria : Proposed limits for environmental regulation’, *Environment International*. The Authors, 86, pp. 140–149. doi: 10.1016/j.envint.2015.10.015.

Bennish, M. L. and Ahmed, S. (2020) ‘Shigellosis’, *Hunter’s Tropical Medicine and Emerging Infectious Diseases*. Elsevier, pp. 492–499. doi: 10.1016/B978-0-323-55512-8.00048-X.

Bottone, E. J. (2010) ‘Bacillus cereus, a volatile human pathogen’, *Clinical Microbiology Reviews*, 23(2), pp. 382–398. doi: 10.1128/CMR.00073-09.

Buckle, J. (2015) ‘Chapter 7 – Infection’, *Clinical Aromatherapy*, (Brooker 2008), pp. 130–167. doi: 10.1016/B978-0-7020-5440-2.00007-3.

Chen, X. *et al.* (2013) ‘Plesiomonas shigelloides infection in Southeast China’, *PLoS ONE*, 8(11). doi: 10.1371/journal.pone.0077877.

Diederen, B. M. W. (2008) ‘Legionella spp. and Legionnaires’ disease’, *Journal of Infection*, 56(1), pp. 1–12. doi: 10.1016/j.jinf.2007.09.010.

Drancourt, M. (2010) ‘Acute diarrhea’, *Infectious Diseases: Third Edition*. Mosby, 1, pp. 381–388. doi: 10.1016/B978-0-323-04579-7.00035-6.

Galloway, D. and Cohen, M. B. (2021) ‘Infectious Diarrhea’, *Pediatric Gastrointestinal and Liver Disease*. Elsevier, pp. 398-415.e5. doi: 10.1016/B978-0-323-67293-1.00038-4.

Geme, J. W. St. and Rempe, K. A. (2018) *Haemophilus influenzae*. Fifth Edit, *Principles and Practice of Pediatric Infectious Diseases*. Fifth Edit. Elsevier Inc. doi: 10.1016/B978-0-323-40181-4.00172-9.

Janda, J. M. and Abbott, S. L. (2010) ‘The genus Aeromonas: Taxonomy, pathogenicity, and infection’, *Clinical Microbiology Reviews*, 23(1), pp. 35–73. doi: 10.1128/CMR.00039-09.

Jha, A. K., Bais, H. P. and Vivanco, J. M. (2005) ‘Enterococcus faecalis mammalian virulence-related factors exhibit potent pathogenicity in the Arabidopsis thaliana plant model’, *Infection and Immunity*, 73(1), pp. 464–475. doi: 10.1128/IAI.73.1.464-475.2005.

Li, J. W., Wyllie, R. M. and Jensen, P. A. (2021) ‘A Novel Competence Pathway in the Oral Pathogen Streptococcus sobrinus’, *Journal of dental research*. J Dent Res, 100(5), pp. 542–548. doi: 10.1177/0022034520979150.

Murray, A. K. *et al.* (2020) ‘The “selection end points in communities of bacteria” (Select) method: A novel experimental assay to facilitate risk assessment of selection for antimicrobial resistance in the environment’, *Environmental Health Perspectives*, 128(10), pp. 107007-1-107007–10. doi: 10.1289/EHP6635.

Payeur, J. B. (2014) ‘Mycobacterium’, *Encyclopedia of Food Microbiology: Second Edition*, 2, pp. 841–853. doi: 10.1016/B978-0-12-384730-0.00229-9.

Qureshi, S. and Qamar, F. N. (2020) ‘Miscellaneous Bacterial Enteritides’, *Hunter’s Tropical Medicine and Emerging Infectious Diseases*. Elsevier, pp. 512–517. doi: 10.1016/B978-0-323-55512-8.00051-X.

Smith, J. L. (2014) *Hafnia, The Genus*. Second Edi, *Encyclopedia of Food Microbiology: Second Edition*. Second Edi. Elsevier. doi: 10.1016/B978-0-12-384730-0.00150-6.

Soumana, I. H., Linz, B. and Harvill, E. T. (2017) ‘Environmental origin of the genus Bordetella’, *Frontiers in Microbiology*, 8(JAN), pp. 1–10. doi: 10.3389/fmicb.2017.00028.

Turnbull, P. C. B. (1996) ‘Bacillus’, *Biotechnology: Second, Completely Revised Edition*. University of Texas Medical Branch at Galveston, 1–12, pp. 367–400. Available at: https://www.ncbi.nlm.nih.gov/books/NBK7699/ (Accessed: 6 July 2021).

Wanger, A. *et al.* (2017) *Overview of Bacteria*, *Microbiology and Molecular Diagnosis in Pathology*. doi: 10.1016/b978-0-12-805351-5.00006-5.

Wong, D. *et al.* (2017) ‘Clinical and pathophysiological overview of Acinetobacter infections: A century of challenges’, *Clinical Microbiology Reviews*, 30(1), pp. 409–447. doi: 10.1128/CMR.00058-16.

Wu, M. and Li, X. (2014) *Klebsiella pneumoniae and Pseudomonas aeruginosa*, *Molecular Medical Microbiology: Second Edition*. Elsevier Ltd. doi: 10.1016/B978-0-12-397169-2.00087-1.
